# HIV Drug Therapy in the Americas 16–18 April 2015, Mexico City, Mexico

**DOI:** 10.7448/IAS.18.3.20177

**Published:** 2015-04-16

**Table:** 

Infection	Number of patients tested	Number of patients with reactive test	Prevalence (%)
HIV	615	297	48
HCV	197	6	3
HBV	232	15	6.5
Syphilis	230	45	19.6
