# Supplementary material for: HIV Drug Therapy in the Americas 16–18 April 2015, Mexico City, Mexico
Source: J Int AIDS Soc. 2015 Apr 16;18(3Suppl 2):20177. doi: 10.7448/IAS.18.3.20177 (PMC4401943; doi:10.7448/IAS.18.3.20177)
Supplement: Related texts

# Sub article: Immune activation in treated HIV infection: does it matter?

**DOI:** 10.7448/IAS.18.3.20100

**Published:** 2015-04-16

**Authors:** Mike Lederman

**Affiliations:** Case Western Reserve University School of Medicine and University Hospitals of Cleveland, Cleveland, OH, USA

## Abstract

Antiretroviral therapies have proved life-saving in HIV infection, dramatically reducing morbidity and mortality. With longer survival, morbidities and mortalities in HIV infection are increasingly similar to the morbidities and mortalities associated with ageing. In treated HIV infection, the risk of these morbidities and mortalities is linked to immune activation, inflammation and coagulation indices. And in persons with treated HIV infection, failure to restore circulating CD4 T cell numbers is associated with a greater risk of morbidities and mortalities as well as to heightened levels of inflammation and coagulation. The drivers of immune activation, inflammation and coagulation in treated HIV infection are incompletely defined and could be related to sustained low levels of viral replication in tissues, to translocation of microbial products across a damaged gut mucosa, to replication of co-pathogens such as cytomegalovirus, to increased levels of inflammatory lipids, or to homeostatic responses to lymphocytopenia that may drive expansion of CD8 T cell numbers. We present here models that link inflammation and coagulation to morbid outcomes as well as to the pathogenesis of CD4 T cell restoration failure and CD8 T cell expansion.

# Sub article: Heterogeneity is your friend: using data to drive performance improvement in HIV programs

**DOI:** 10.7448/IAS.18.3.20101

**Published:** 2015-04-16

**Authors:** Sergio Bautista-Arredondo, Stefano Bertozzi, Sandra Sosa-Rubi

**Affiliations:** 1 National Institute of Public Health, Cuernavaca, Mexico; 2 Health Policy and Management, University of California Berkeley, School of Public Health, Berkeley, CA, USA

## Abstract

Although the performance of HIV prevention and treatment programs varies considerably among countries, the variability within any one country is typically much greater than is the variability among countries. Even within an individual health system with relatively homogeneous conditions for its clinics (resource constraints, human resource system, professional training standards, compensation, incentives, etc.), there will typically be very heterogeneous performance across clinics. We have the opportunity to analyse existing heterogeneity to both learn about what is driving good and poor performance as well as to create incentives for clinics and providers to learn from and emulate the top performers. We will use examples from Mexico and Africa to show how much heterogeneity exists, discuss cases in which revealing that heterogeneity has driven performance improvement and speculate on how such approaches could be implemented more broadly in Latin America.

# Sub article: Pre-exposure prophylaxis: an update

**DOI:** 10.7448/IAS.18.3.20102

**Published:** 2015-04-16

**Authors:** Roy Gulick

**Affiliations:** Division of Infectious Diseases, Weill Cornell Medical College, Cornell University, New York, NY, USA

## Abstract

Among a number of HIV prevention approaches, pre-exposure prophylaxis (PrEP) is the strategy of administering antiretroviral drug(s) to HIV-positive people at risk for HIV infection to decrease their risk of acquiring HIV infection. With 28 approved antiretroviral drugs, tenofovir with or without emtricitabine emerged as the leading candidate(s) for PrEP, with demonstrated potency, safety, tolerability and convenience. Randomized, placebo-controlled studies of daily PrEP with tenofovir/emtricitabine in gay men (iPREX) and tenofovir with or without emtricitabine in the negative member of serodiscordant couples (Partners PrEP) demonstrated efficacy in reducing the risk of HIV infection and led to the US Food and Drug Administration approval of tenofovir/emtricitabine for PrEP; this was supported by additional data from the TDF-2 study. Randomized studies in British gay men and Thai injection drug users also demonstrated the efficacy of daily PrEP. Two follow-up studies in African women (FEM-PrEP and Voice) failed to show a benefit, but adherence rates were less than 40%. In all of these studies, adverse event rates were low, drug resistance was very rare and there was no evidence of behavioural disinhibition. More recently, the Ipergay study showed PrEP was effective when given “on demand” – before and after sex, rather than daily. Additional studies of intermittent PrEP dosing are underway. Also, additional antiretroviral agents for PrEP currently are in clinical trials including maraviroc and the long-acting parenteral formulations of rilpivirine and cabotegravir. Current US and WHO guidelines recommend PrEP for people at high risk for HIV infection, including gay men, heterosexuals and injection drug users.

# Sub article: What will it take to end the AIDS epidemic?

**DOI:** 10.7448/IAS.18.3.20103

**Published:** 2015-04-16

**Authors:** Carlos del Rio

**Affiliations:** Hubert Department of Global Health, Rollins School of Public Health of Emory University, Emory Center for AIDS Research, Atlanta, GA, USA

## Abstract

In the past four years, unprecedented breakthroughs in HIV prevention, treatment and research have occurred. Particularly notable are the results of HPTN 052 which demonstrated that effective antiretroviral therapy of HIV-positive individuals dramatically cuts HIV transmission and the results of several studies demonstrating the impact on HIV acquisition of pre-exposure prophylaxis. These advances have made many realize that we can end the AIDS epidemic by 2030. However, a myriad of challenges will need to be overcome in order to achieve this goal. The first challenge is financial; although the mobilization of resources to treat HIV has been unprecedented, we are far from the necessary resources needed to treat over 90% of those infected. The second challenge is in implementation, in most of the world weak healthcare systems that are understaffed struggle to manage their current patient load, let alone the millions of additional patients who will require treatment. In addition, in order to ensure timely linkage to care after diagnosis and retention in care, healthcare systems will require to be reengineered and novel interventions will need to be developed. Furthermore, laboratory monitoring continues to be limited in most countries. The third challenge is persistent stigma and discrimination that has prevented many who are at risk or infected from accessing care. Finally, there is the challenge of political and societal will. It is theoretically possible to end the AIDS epidemic, but it will happen unless we dramatically change the way we are currently doing things.

# Sub article: HIV and vulnerable populations

**DOI:** 10.7448/IAS.18.3.20104

**Published:** 2015-04-16

**Authors:** Chris Beyrer

**Affiliations:** Johns Hopkins Training Program in HIV Epidemiology and Prevention Science, Baltimore, MD, USA

## Abstract

Key populations share high burdens of HIV infection and low access to HIV services, and include people who inject drugs (PWID), sex workers of all genders, gay and other men who have sex with men (MSM), transgender women who have sex with men, and prisoners and detainees. These communities now account for an estimated 50% of new HIV infections worldwide and are at the centre of the HIV epidemic zones where HIV is still expanding: Eastern Europe and Central Asia, and the Middle East and North Africa. These populations have lower HIV treatment access in many settings and are excluded, or exclude themselves, from life-saving treatment and related services. Little data is available on the continuum of care for key populations, but it will be essential to better understand the barriers to successful testing, linkage, retention and successful viral suppression for these individuals and communities if HIV control is to be achieved. Advances and approaches which could improve the continuum of care for key populations will be reviewed.

# Sub article: Health care provision for transgender people in developing countries: the experience of the first transgender health clinic in Mexico

**DOI:** 10.7448/IAS.18.3.20105

**Published:** 2015-04-16

**Authors:** Esmeralda Román, Hamid Vega-Ramirez, Jeremy Cruz, Jesús Casillas-Rodríguez, Andrea González-Rodríguez, Pablo Belaunzarán-Zamudio, Juan Sierra-Madero

**Affiliations:** 1 Health Care Center for Transgender People, Clínica Especializada Condesa, Mexico City, Mexico; 2 Salud Mental, Clínica Especializada Condesa, Mexico City, Mexico; 3 Dirección Médica, Clínica Especializada Condesa, Mexico City, Mexico; 4 Dirección, Programa de VIH/SIDA de la Ciudad de México, Mexico City, Mexico; 5 Coordinación de Investigación, Programa de VIH/SIDA de la Ciudad de México, Mexico City, Mexico; 6 Infectious Diseases, Instituto Nacional de Ciencias Médicas y Nutrición “Salvador Zubirán”, Mexico City, Mexico

## Abstract

**Introduction:**

Transgender people have specific health problems and challenges related to stigma, hormonal administration, sexual risk behaviour and application of body-modifying substances. There is limited experience on the provision of integral health care services for transgender people, particularly in developing countries. We describe the experience providing health care services for transgender people at Clínica Especializada Condesa (CEC) in Mexico City.

**Materials and methods:**

The Health Care Center for Transgender People Program started in 2009. It is the first specialized Clinic in Transgender Health in Mexico. We describe the model of integral health care for transgender people and basal information on the beneficiary population.

**Results:**

Primary health care is provided by one endocrinologist aided by two nurse practitioners, three psychiatrist, three psychologists, one gynaecologist, one radiologist and a specialised clinical laboratory. Internal Medicine specialists provide HIV/AIDS care for those HIV-positive. All patients receive Voluntary Counselling and Testing (VCT) and mental health care services. These comprise an extensive evaluation for gender life-experience, drug abuse and psychopathologies. Cross-sexual hormonal therapy (HT) is provided for free in selected individuals, according to pre-established criteria based in life experience as their cross-sexual role, mental health, age, co-morbidities and HIV status and antiretroviral therapy (ART) adherence. There are 1187 patients registered in the clinic, 889 whom actively are currently receiving care. The mean age is 31 (16–68) years old. There are 1004 male-to-female (MtoF) (84.58%) persons, and 65.63% (*n=*659) receive HT. A total of 615 (52%) have been tested for HIV of whom 297 (48%) are HIV-positive.

A total of 191 (64%) of HIV-positive patients concomitantly receive HT and ART. Among those HIV-positive and receiving ART (*n=*252), the majority (*n*=220, 87%) have achieved complete viral suppression. The program faces two main challenges: frequent changes in primary care provider and inconsistent supply of HT. Changes in HT regime due to unavailability of specific compounds are frequent (93%), and almost all patients have changed HT for this reason.

**Conclusions:**

The provision of integral health care services for transgender people in an urban setting of middle-income country is feasible. Limited resources and personnel rotation are the main challenges. Provision of free HT in selected cases appears to be safe and may contribute to retention in care.

# Sub article: Barriers to care and reproductive choices in Latin American HIV+ women: a subanalysis of the ELLA Study

**DOI:** 10.7448/IAS.18.3.20106

**Published:** 2015-04-16

**Authors:** Gabriela Bugarin, César Rivera, Isabel Cassetti, Patricia Vasquez, José Valdez Madruga, Jean van Wyk, Marisol Martinez-Tristani

**Affiliations:** 1 Abbvie Inc., Medical Affairs, North Chicago, IL, USA; 2 Infectious Diseases, Hospital General de México, México City, Mexico; 3 Infectious Diseases, Helios Salud, Buenos Aires, Argentina; 4 Infectious Diseases, Hospital San Juan de Dios, Santiago de Chile, Chile; 5 Infectious Diseases, Centro de Referência e Treinamento DST/AIDS, São Paulo, Brazil

## Abstract

**Introduction:**

Global prevalence of HIV-1 is estimated at 35 million; >50% of those infected are women. Despite availability of effective antiretroviral therapy (ART), many HIV-positive individuals are not in care; women have greater difficulty accessing HIV care than men. The ELLA study is a cross-sectional, multi-country, non-interventional epidemiological study to investigate the population, disease characteristics, barriers to care, quality of life (QOL) and reproductive choices for women living with HIV. We describe results of women included in ELLA from the Latin American region (LA).

**Materials and methods:**

HIV-1-positive females ≥18 years were enrolled using a non-random sequential sampling frame in four global geographic regions: Western Europe and Canada, LA, Central/Eastern Europe and Asia. Eligible women completed self-administered Barriers to Access to Care Scale (BACS) and other health status questionnaires. Patients rated each of the 12 BACS items using a four-point Likert scale (1=“No problem at all” to 4=“Major problem”). Questionnaires with ≥6 items completed were included in the analysis, mean score >2.0 was considered a significant barrier to access to care.

**Results:**

A total of 519 women participated in ELLA from the LA region (total *N*=1922). For these women, mean age was 42.2 years and 96.7% acquired HIV through sexual contact. A total of 54.4% had been diagnosed with HIV >5 years and 87.3% were currently on ART. Recent CD4 count was >500/mm^3^ in 48%, and most recent viral load was <50 c/ml in 52.2%. More than 8 years formal education was reported by 69.3%, 45.9% lived with a partner/husband, and 55.3% were employed. Mean overall BACS score was 2.2 across all 12 individual items. Highest barriers to access to care were related to community stigma (mean score 3.1), lack of personal resources (mean score 2.5). Women had an average of 1.9 children (range 0–12) with 17% indicating a desire to have more children (32% in women <35 years). Birth control strategies were mainly based on female surgery (33.3%), male condom use (68.3%), 19.7% of women reporting abstinence.

**Conclusions:**

The majority of HIV+ women in LA included in ELLA were receiving ART, approximately half reported to have undetectable viral loads and normal CD4 cell counts. Barriers to access to care remain high, particularly community stigma and personal resources. Special attention on reproductive health counselling, should be considered.

# Sub article: Most important infectious comorbidities in the region and how to manage them

**DOI:** 10.7448/IAS.18.3.20107

**Published:** 2015-04-16

**Authors:** Omar Sued

**Affiliations:** Fundación Huesped, Buenos Aires, Argentina

## Abstract

Despite the advance that highly active antiretroviral therapy (HAART) represents for the prognosis of HIV infection, opportunistic infections (OIs) continue to be a significant cause of morbidity and mortality in HIV-positive patients. Lack of access, late diagnosis, leaks in the HIV cascade of care, lack of treatment adherence and failure put patients at risk of developing OIs and dying from AIDS.

Nevertheless, HAART introduced changes in the prevalence of some IOs. Currently, the frequency of severe cytomegalovirus (CMV) retinal disease, *Mycobacterium avium-intracellulare* (MAI) and latent membrane protein (LMP) is lower than in the early years of the epidemic. *Pneumocystis jiroveci*, tuberculosis and toxoplasmic encephalitis are the most common opportunistic infections. Disseminated mycoses are very frequent in the region, for example, in countries such as Brazil or Colombia, cryptococcal meningitis is even more frequent than toxoplasmic encephalitis. Some diseases are limited to our continent. This is the case of Histoplasmosis, whose diagnosis might be difficultin areas where urinary antigen is not available. The diagnosis and management of Chagas disease, a parasitic disease endemic only in Latin America, might be also challenging. Viral infections are more frequent in HIV-positive patients (HAV, HBV, HCV, HPV, CMV and herpes). Some can be easily prevented through vaccines (HAV, HBV, HPV), and some are associated with the development of cancer (HBV, HPV, HHV8, EBV). Finally, an increased trend in sexually transmitted diseases is being observed among HIV-positive individuals. A summary of the current status of the most frequent infections and guidance on the diagnostic, specific treatment and antiretroviral treatment will be presented.

# Sub article: Managing tuberculosis and HIV

**DOI:** 10.7448/IAS.18.3.20108

**Published:** 2015-04-16

**Authors:** Timothy R Sterling

**Affiliations:** Vanderbilt Tuberculosis Center, Vanderbilt University School of Medicine, Nashville, TN, USA

## Abstract

The global epidemics of tuberculosis and HIV are closely linked. Among persons with latent *Mycobacterium tuberculosis* infection, HIV is the strongest risk factor for progressing to active, infectious tuberculosis. Although antiretroviral therapy substantially decreases the risk of developing tuberculosis, it may also unmask previously unrecognized tuberculosis in the setting of immune reconstitution. Concomitant treatment of tuberculosis and HIV can be complicated by drug-drug interactions and immune reconstitution inflammatory syndrome (IRIS). The timing of antiretroviral therapy initiation in relation to initiation of anti-tuberculosis therapy is also important. Treatment of latent *M. tuberculosis* infection in HIV-infected persons decreases tuberculosis risk in addition to the benefit provided by antiretroviral therapy, though the optimal duration of preventive therapy may vary according to local tuberculosis incidence.

# Sub article: Time to HAART initiation after diagnosis and treatment of opportunistic infections in patients with AIDS in the CCASAnet Cohort

**DOI:** 10.7448/IAS.18.3.20109

**Published:** 2015-04-16

**Authors:** Brenda Crabtree Ramírez, Yanink Caro-Vega, Bryan Shepherd, Beatriz Grinsztejn, Marcelo Wolff, Claudia Cortés, Denis Padgett, Gabriela Carriquiry, Valeria Fink, Pablo Belaunzarán-Zamudio, Karu Jayathilake, Anna Person, Catherine McGowan, Juan Sierra-Madero

**Affiliations:** 1 Infectious Diseases, Instituto Nacional de Ciencias Médicas y Nutrición, Salvador Zubirán, Mexico City, Mexico; 2 VCCC, Vanderbilt University, Nashville, TN, USA; 3 Clinical Research Laboratory at IPEC, Instituto de Pesquisa Clinica Evandro Chagas Fiocruz, Rio de Janeiro, Brazil; 4 Infectious Diseases, Universidad de Chile, Hospital San Borja Arriarán, Santiago, Chile; 5 Infectious Diseases, Instituto Hondureño de Seguro Social y Hospital Escuela, Tegucigalpa, Honduras; 6 Infectious Diseases, Intituto de Medicina Tropical Alexander von Humboldt, Lima, Peru; 7 Infectious Diseases, Fundación Huésped, Buenos Aires, Argentina; 8 Infectious Diseases, Vanderbilt University, Nashville, TN, USA

## Abstract

**Introduction:**

Since 2009, earlier initiation of highly active antiretroviral therapy (HAART) after an opportunistic infection (OI) has been recommended based on lower risks of death and AIDS-related progression found in clinical trials. Delay in HAART initiation after OIs may be an important barrier to successful outcomes in patients with advanced disease. Timing of HAART initiation after an OI in “real life” settings in Latin America has not been evaluated.

**Methods:**

All CCASAnet patients >18 years of age at enrolment, from 2001 to 2013, who had an OI before HAART initiation were included. Patients were divided in an Early HAART group (those initiating within four weeks of an OI) and a Delayed HAART group those initiating more than four weeks after an OI. Patients with cancer or cryptococcal infection were excluded. Patients with more than one OI were included using the first OI reported only. Calendar trends in the proportion of patients in the Early HAART group (before and after 2009) were estimated by site and for the whole cohort. Factors associated with Early HAART initiation were estimated with logistic regression.

**Results:**

A total of 20,148 patients were included; 1558 patients had an OI before HAART initiation and were included in the analysis: 207 from Argentina, 746 from Brazil, 322 from Chile, 112 from Honduras and 171 from Mexico. The proportion of patients who started treatment within four weeks of the OI was statistically different between sites (*p*<0.001). Median time since all OIs and non-TB OIs to HAART initiation decreased from 41 (IQR: 20–85) days before 2009 to 30 (IQR: 14–50) after 2009 (Figure 1). Factors associated with Early HAART group were CD4+ at HAART initiation <200 cell/mm^3^ (*p*<0.001), non- tuberculosis OI (*p*<0.001), site and year of initiation (before 2009; *p*<0.001).

**Discussion:**

The time from diagnosis of an OI to HAART initiation has decreased in this Latin American Cohort coinciding with the publication of evidence of its benefit. We found important heterogeneity between sites which may reflect differences in clinical practices, local guidelines and access to HAART. The impact of the timing of HAART initiation after OI on patient survival on this “real life” context needs further evaluation.

**Abstract O123–Figure 1:** Median time since all OIs and non-TB OIs.

# Sub article: Treatment for HIV: new drugs and strategies

**DOI:** 10.7448/IAS.18.3.20110

**Published:** 2015-04-16

**Authors:** Daniel Kuritzkes

**Affiliations:** Division of Infectious Disease, Brigham Women's Hospital, Harvard Medical School Boston, MA, USA

## Abstract

Combination antiretroviral therapy (ART) has reduced dramatically morbidity and mortality from HIV/AIDS worldwide and has the potential to halt HIV transmission. The availability of potent, well-tolerated, convenient regimens – several of which are available as single-tablet regimens – and the demonstration that ART substantially reduces the risk of transmission to uninfected partners has greatly increased enthusiasm for ART. Novel regimens include those based on newer agents approved over the last few years, including the non-nucleoside reverse transcriptase inhibitor (NNRTI) rilpvirine, and the novel integrase strand-transfer inhibitors (INSTIs) elvitegravir and dolutegravir. In addition, the development of cobicistat as an alternative to ritonavir for boosting protease inhibitors is leading to a greater number of fixed-dose combinations of boosted protease inhibitors. An alternative pro-drug of tenofovir, tenofovir alafenamide, achieves higher intracellular concentrations of the active moiety, tenofovir diphosphate, with substantially lower plasma levels of tenofovir, allowing the delivery of more active drug with lower dosing. Several novel agents within existing drug classes have entered clinical trials; drugs directed against novel targets are also in development. Long-acting injectable agents currently in development have the potential to transform treatment and make directly observed therapy an achievable goal. In addition, broadly neutralizing antibodies and a CD4-Ig/CCR5 peptide fusion protein may have a role in HIV therapeutics.

# Sub article: Long term outcomes (10 years) of a cohort of AIDS patients on ART in Haiti

**DOI:** 10.7448/IAS.18.3.20111

**Published:** 2015-04-16

**Authors:** Jean William Pape

**Affiliations:** 1 Division of Infectious Diseases, Centre for Global Health, Weill Cornell Medical College, Cornell University, New York, NY, USA; 2 Les Centres GHESKIO, Port-au-Prince, Haiti

## Abstract

The Haitian Study Group on Kaposi Sarcoma and Opportunistic Infections (GHESKIO) in Port-au-Prince, Haiti, is the first AIDS prevention and treatment centre in the world. It is also one of the largest AIDS care centres in the Americas. Highly active antiretroviral therapy (ART) was made available in Haiti in 2003 and 2004 with the support respectively of the Global Fund against AIDS, TB and Malaria (GATM) and PEPFAR. From February 2003 to February 2015, 17,029 patients, 13 years old and older, have been placed on ART at GHESKIO-INLR, one of the two main GHESKIO centres in Port-au-Prince. GHESKIO conducted an observational cohort study on the outcomes of a non-research cohort of 1463 patients 13 years old and older starting their first ART regimen and followed for 10 years from February 6, 2013, to February 5, 2015. The primary endpoint was all-cause mortality. The probability of mortality after ART initiation was estimated using Kaplan-Meir methods, censoring subjects lost to follow-up at the time of their last visit. Median age for the entire cohort was 38 years old (IQT 32–45); 54% were females; the median entry CD4 counts was 130/mm^3^ (51–217); and 40% had AIDS by WHO criteria. The overall probability of survival at one year, five years and ten years was respectively 82, 72 and 50%. Mortality was 23% during the period. It was highest during the first three months and lowest from 5 to 10 years. The lost to follow-up rate during the 10-year period was 15% and was more evenly distributed. Factors associated with mortality and lost to follow-up will be presented.

# Sub article: ARV regimens after first line failure: current status and remaining questions

**DOI:** 10.7448/IAS.18.3.20112

**Published:** 2015-04-16

**Authors:** Juan Sierra-Madero

**Affiliations:** National Institute of Medical Sciences and Nutrition, Mexico City, Mexico

## Abstract

A significant proportion of patients (approximately 25% in five years according to recent data from the region) require a second-line regimen because of failure of a first one. WHO guidelines suggest using lopinavir/ritonavir plus tenofovir/emtricitabine after first-line failure of an NNRTI-based regimen. Clinical trials exploring the efficacy and safety of the WHO recommended regimen compared to nucleoside-sparing regimens using integrase inhibitors have been conducted in recent years. The second-line study demonstrated non-inferiority of raltegravir/lopinavir/r compared to the standard WHO regimen at 96 weeks. In the Earnest study, a composite endpoint defined as “good HIV disease control” was reached in a similar proportion of patients treated with the WHO recommended regimen and those treated with raltegravir plus lopinavir/r. In both studies, the frequency of tenofovir use in the initial regimen was low (approximately 20%). Nucleoside-sparing regimens were associated with lower bone loss and higher limb fat gain in the second line trial. Other studies using the newer integrase inhibitor dolutegravir with or without nucleos(t)ides are currently being conducted or planned. The long-term efficacy, tolerability and cost-effectiveness of different second-line regimens as well as predictors of success both in clinical trials and “real life situations” remain to be evaluated. The efficacy of newer regimens, including single tablet combinations, the role of resistance testing to guide second-line treatment and second-line regimens used after a first-line boosted PI regimen are unanswered questions.

# Sub article: Quantifying drug adherence to antiretroviral therapy: what is new and what could help us?

**DOI:** 10.7448/IAS.18.3.20113

**Published:** 2015-04-16

**Authors:** Jose Castillo-Mancilla

**Affiliations:** Division of Infectious Diseases, School of Medicine, University of Colorado Denver, Aurora, Denver, CO, USA

## Abstract

Antiretroviral exposure is critical to achieve viral suppression in HIV-positive patients and to prevent transmission to HIV-negative individuals. It is directly linked to individual host factors which include age, weight, diet and genetics. However, the main factor impacting long-term drug exposure is drug adherence. Although modern regimens are more forgiving to lower levels of adherence due to their higher potency and more favourable pharmacokinetics, adherence remains the major predictor of HIV outcomes. Unfortunately, quantifying adherence is difficult due to the inherent limitations of the adherence measures currently available in routine clinical practice and research settings. This indicates the need for new and objective measures of antiretroviral exposure that can integrate adherence and pharmacokinetics and provide additional information to what we obtain from the currently available methods. This presentation will focus on some of the new measures that have been recently developed to measure and monitor adherence to antiretrovirals and on the potential approaches that could lead to their utilization in the clinical and research settings.

# Sub article: Cancers and HIV infection: clinical care

**DOI:** 10.7448/IAS.18.3.20114

**Published:** 2015-04-16

**Authors:** Patricia Volkow

**Affiliations:** National Institute of Cancer, Mexico City, Mexico

## Abstract

Malignancies were hallmarks of the AIDS epidemic. Kaposi's sarcoma (KS), the first to be identified, was first described among MSM in 1981 as an enigmatic and unpredictable disease and its management has created great controversy. The human herpes virus-8 (HHV-8), discovered in 1994, is present in all KS lesions, later demonstrated that level of HHV-8 viremia correlated with the severity of the disease and identification of high levels of inflammatory cytokines. Additionally, as many as 80% of KS patients achieve complete remission solely with highly active antiretroviral therapy (HAART), and the occurrence of immune recovery syndrome with exacerbation of lesion in patients starting HAART and the polyclonality of lesions create the need to understand KS as an angioproliferative disease caused by an infectious agent mediated by cytokines. In its management, recovery of immunosuppression is essential as is the treatment of co-infections and avoidance of myelosuppressive therapy. Non-Hodgkin lymphoma has become the most frequent malignancy seen in HIV patients; it requires standard chemotherapy regimens and HAART along with prophylaxis for opportunistic infections while on chemotherapy. The HPV-associated neoplasia, incorporated as an AIDS-defining event in 1993, constitutes a major burden of morbidity in HIV women in countries with high prevalence of oncogenic HPV virus infection. In Mexico, 21% have been diagnosed with a high-grade lesion and 14% with cervical cancer. Recently, higher risk for vulvar and vaginal cancer has also been recognized with the increase survival of women on HAART, and thus the need for colposcopic evaluation of the genital tract and not only PAP smear screening for HPV lesions is required.

# Sub article: HIV and ageing populations

**DOI:** 10.7448/IAS.18.3.20115

**Published:** 2015-04-16

**Authors:** Rafael Campo

**Affiliations:** Division of Infectious Diseases, University of Miami School of Medicine, Miami, FL, USA

## Abstract

HIV infection is rapidly becoming an affliction of older individuals. It is estimated that sometime in 2015, over 50% of HIV-positive individuals in the United States will be over 50 years of age, and this phenomenon is occurring all over the world. It is a function of both patients living longer and older individuals becoming infected more frequently than in the past. Multiple factors are associated with the widely held misconception by the general public and healthcare workers that HIV continues to be a disease of younger people. Thus, more aggressive efforts need to be directed at testing, diagnosing and bringing into care older patients. However, we also need to develop more effective educational messages for those uninfected but at-risk individuals who mistakenly assume that they are protected from HIV infection because they are “old.” With this ageing of the population, it is becoming clear that specific challenges have arisen. While older patients frequently exhibit better adherence to antiretroviral therapy and respond quite well to it, their immune recovery is slower, they are at higher risk of progressing to AIDS and their mortality is higher. In addition to this, the normal ageing process seems to be accelerated by HIV infection itself, and it has become clear that the morbidities associated with advancing age are both more frequent and advance faster than in non-infected, age-matched individuals. Thus, more effective screening, prevention and management programs are needed for cardiovascular, metabolic, neurocognitive, renal, hepatic, hematologic and oncologic diseases of an ageing HIV-positive population.

# Sub article: A review of key data presented/published since the last HIV in the Americas Congress

**DOI:** 10.7448/IAS.18.3.20116

**Published:** 2015-04-16

**Authors:** Anton Pozniak

**Affiliations:** HIV Services, Chelsea & Westminster Hospital, London, UK

## Abstract

It has been an important year for pre-exposure prophylaxis (PrEP). Further analysis of the Pre-exposure Prophylaxis Initiative (iPrEx) study showed that taking PrEP of at least four tablets weekly provides almost complete protection against HIV infection. Then came the PROUD and Ipergay studies showing 86% protection in high-risk men who have sex with men (MSM) who took continuous or intermittent (on demand) truvada. Now the issue will be about implementation of such successful strategies worldwide. For those patients who cannot take efavirenz first line what is the best strategy? A study of other initial therapies compared raltegravir vs boosted darunavir vs boosted atazanavir (ACTG 5257). Overall, using intent-to-treat (ITT), all regimens were of similar efficacy but if switching for tolerability or toxicity was taken into account, then raltegravir was better than the boosted proteases. Nucleoside-free or -limiting approaches were also prominent with the 96-week data from NEAT 001 study of darunavir and ritonavir plus either raltegravir or nukes. The combination did not perform well when the CD4 was <200 cells/mm. The 48-week GARDEL study of the two drug regimen of lopinavir and ritonavir plus lamivudine showed similar efficacy as triple therapy. This same nuke-limiting strategy was used in maintenance treatment with the SALT study, atazanavir and ritonavir plus lamivudine and the open label extension (OLE) study of lopinavir and ritonavir plus lamivudine. A nearly twofold increased risk for suicidality in efavirenz-treated patients was found by a *post hoc* analysis of several ACTG studies which has not been seen in the cohort studies. More controversy has been fuelled by the recent North American-AIDS Cohort Collaboration on Research and Design (NA-ACCORD) data linking abacavir and myocardial infarct although the most restricted analysis was statistically non-significant. Interferon-free therapy for hepatitis C is becoming a reality with different combinations of oral drugs such as sofosbuvir plus simeprevir or with ledipasvir or the Abbvie triple combination. The issues still remain regarding cost as well as how to treat the most difficult patients. The big opportunistic infection (OI) trial was the COAT study confirming that early HIV treatment was associated with a worse outcome in cryptococcal meningitis. Finally cure – only one patient has been cured of HIV the others who were thought to have been, the Boston two stem cell transplant patients and the Mississippi baby – all relapsed. A TLR7 agonist is going into human trials so the field is moving slowly forward.

# Sub article: Progress of the dual elimination of mother to child transmission of HIV and congenital syphilis in Latin America and the Caribbean

**DOI:** 10.7448/IAS.18.3.20117

**Published:** 2015-04-16

**Authors:** Mónica Alonso González

**Affiliations:** Pan American Health Organization (PAHO), Washington, DC, USA

## Abstract

**Introduction:**

Countries in the Americas committed to eliminating mother to child transmission (MTCT) of HIV and syphilis by 2015. Ambitious goals were set for virtual elimination of both conditions by 2015 through a robust public health approach. The Pan American Health Organization (PAHO) monitors country and regional progress towards elimination. The analysis presented may assist policy-makers and healthcare workers in their efforts to achieve elimination of MTCT of HIV and syphilis in the Americas.

**Methods:**

The presentation will summarize progress towards elimination goals from 2010, using data reported by countries to PAHO on sexual and reproductive health, policies and provision of services, and outcomes regarding paediatric HIV and congenital syphilis cases in the Americas.

**Results:**

The coverage of HIV testing among pregnant women in Latin America and the Caribbean has increased from 62% in 2010 to 74% in 2013, and the estimated coverage of ARV coverage among HIV-positive pregnant women has increased from 59% in 2010 to 93% in 2013. The calculated regional vertical HIV transmission rate has decreased from 18% (14–25%) in 2010 to 5% (2–23%) in 2013. Progress in the area of syphilis has been less pronounced, with the coverage of syphilis testing among pregnant women remaining stable in 20 reporting countries at around 80%. Syphilis treatment coverage for pregnant women remains largely unreported. Based on 2013 data, at least seven countries and territories may have achieved the dual EMTCT targets, with at least another eight approaching the targets. Around 21 countries had insufficient information to ascertain progress, of which several may also have achieved dual elimination.

**Conclusions:**

The progress in the region of the Americas illustrates how political and programmatic efforts can result in rapid progress and major public health gains.

# Sub article: Sofosbuvir and ribavirin therapy for the treatment of HIV/HCV co-infected patients with HCV GT1-4 infection: the PHOTON-1 and -2 Trials

**DOI:** 10.7448/IAS.18.3.20118

**Published:** 2015-04-16

**Authors:** Jorge Santana, Juergen Rockstroh, Massimo Puoti, Maribel Rodriguez-Torres, Douglas Dieterich, Anuj Gaggar, Liyun Ni, Benedetta Massetto, Evguenia Svarovskaia, Diana Brainard, Mani Subramanian, John McHutchison, Susanna Naggie, Chloe Orkin, Jean-Michel Molina, Mark Sulkowski

**Affiliations:** 1 School of Medicine, University of Puerto Rico, San Juan, Puerto Rico; 2 Department of General Internal Medicine, University Hospital of Bonn, Bonn, Germany; 3 Division of Infectious Diseases, AO Ospedale Niguarda Ca’ Granda, Milano, Italy; 4 SJB School of Medicine, Fundacion De Investigacion, San Juan, Puerto Rico; 5 Mount Sinai School of Medicine, New York, NY, USA; 6 Gilead Sciences, Clinical Research, Foster City, CA, USA; 7 Gilead Sciences, Biostatistics, Foster City, CA, USA; 8 Gilead Sciences, Biology, Foster City, CA, USA; 9 Duke Clinical Research Institute, Durham, NC, USA; 10 Barts Health NHS Trust, London, UK; 11 Department of Infectious Diseases, Saint-Louis Hospital, Paris, France; 12 School of Medicine, Johns Hopkins University, Baltimore, MD, USA

## Abstract

**Introduction:**

Interferon-free treatments for HCV that can be safely administered with antiretroviral therapy (ART) are needed for HIV/HCV co-infected patients. These two studies evaluated the safety and efficacy of sofosbuvir (SOF), a pan-genotypic HCV NS5B inhibitor, with ribavirin (RBV) in individuals co-infected with HIV and HCV genotype (GT) 1–4.

**Methodology:**

In total, 497 HCV-HIV co-infected patients were enrolled in the PHOTON-1 or PHOTON-2 Phase 3 studies to receive SOF 400 mg QD and RBV 1000–1200 mg/day for 12 or 24 weeks based on HCV genotype and prior treatment status. Multiple ART regimens were permitted as were patients with compensated cirrhosis. The primary efficacy endpoint was sustained virologic response 12 weeks after treatment (SVR12); safety assessments included HIV RNA and CD4 cell levels.

**Results:**

Baseline demographics and virologic responses are shown in the table (Table 1). SVR12 rates were 80–91% with the exception of GT3 HCV patients treated with 12 weeks of SOF + RBV (67%). Among 76 patients with cirrhosis, 59 (77%) achieved SVR12. Multivariate analyses of baseline characteristics associated with SVR, by HCV genotype, showed that significant predictors for SVR12 were non-black race and absence of cirrhosis for GT1 patients, and lower HCV RNA level at baseline and a longer treatment duration for GT3 patients. A total of 445 subjects (89.5%) experienced any adverse event (AE) but only 8% had a Grade 3 or 4 AE and 2.5% had an AE resulting in early SOF discontinuation. There was no change in CD4 T-cell percentage during treatment. Among patients suppressed on ART, 1% had HIV virologic breakthrough, though none of these subjects required a change in ART.

**Conclusions:**

HCV GT 1–4 patients co-infected with HIV achieved high rates of SVR12 with an interferon-free, all-oral regimen of SOF + RBV. This pooled analysis from two Phase 3 studies further demonstrates that SOF + RBV treatment was well-tolerated and safely co-administered with multiple ART regimens, and suggests that concurrent HIV-1 infection does not reduce SVR12 rates with sofosbuvir-based regimens.

**Abstract O322–Table 1:** Baseline demographics and virologic responses

	GT 1 TN 24 weeks (*n*=226)	GT 2 TN 12 weeks (*n*=45)	GT 2 TE 24 weeks (*n*=30)	GT 3 TN 12 weeks (*n*=4)	GT 3 TN 24 weeks (*n*=57)	GT 3 TE 24 weeks (*n*=66)	GT 4 TN 24 weeks (*n*=31)
Baseline characteristics							
Male, *n (*%)	193 (85)	36 (80)	29 (97)	34 (81)	38 (67)	52 (79)	24 (77)
Black, *n* (%)	38 (17)	6 (13)	7 (23)	2 (5)	0	1 (2)	1 (3)
IL28B CC genotype, *n* (%)	78 (35)	22 (49)	13 (43)	15 (36)	30 (53)	25 (53)	9 (29)
Cirrhosis, *n* (%)	22 (10)	2 (4)	6 (20)	6 (14)	3 (5)	29 (44)	8 (26)
Log_10_ HCV RNA (IU/mL), mean (SD)	6.5 (0.8)	6.6 (0.6)	6.5 (0.8)	6.6 (0.6)	6.3 (0.7)	6.3 (0.7)	5.9 (0.9)
CD4 T-cell count (cells/µL), mean (SD)	632 (265)	573 (232)	646 (314)	559 (224)	572 (268)	600 (278)	545 (208)
On ART, *n* (%)	221 (98)	39 (87)	29 (97)	39 (93)	57 (100)	62 (94)	30 (97)
SVR12, *n/N* (%)	181/226 (80)	40/45 (89)	27/30 (90)	28/42 (67)	52/57 (91)	58/66 (88)	26/31 (84)

# Sub article: HIV drug resistance in treatment-naïve individuals in Mesoamerica

**DOI:** 10.7448/IAS.18.3.20119

**Published:** 2015-04-16

**Authors:** Santiago Avila-Rios, Claudia García-Morales, Daniela Tapia-Trejo, Carlos Mejía-Villatoro, Juan Pascale, Guillermo Porras-Cortes, Ivette Lorenzana, Elsa Palou, Marvin Manzanero, Gustavo Reyes-Terán

**Affiliations:** 1 Center for Research in Infectious Diseases, National Institute of Respiratory Diseases, Mexico City, Mexico; 2 Infectious Diseases Clinic, Roosevelt Hospital, Guatemala City, Guatemala; 3 Gorgas Memorial Institute for Health Studies, Panama City, Panama; 4 Vivian Pellas Metropolitan Hospital, Managua, Nicaragua; 5 Department of HIV/AIDS, Universidad Nacional Autónoma de Honduras, Tegucigalpa, Honduras; 6 Ministry of Health, Belize, Belize

## Abstract

**Introduction:**

HIV drug resistance (DR) surveillance in antiretroviral treatment (ART)-naïve individuals (pre-ART DR, PDR) is key to maximizing the long-term effectiveness of ART regimens and to ensure sustainability of ART programs. We assessed HIV PDR in Mexico and Central America.

**Materials and methods:**

ART-naïve individuals from Mexico (*n*=1634), Guatemala (*n*=1283), Panama (*n*=277), Nicaragua (*n*=236), Honduras (*n*=341) and Belize (*n*=117) were enrolled from October 2010 to December 2014. Plasma HIV pol sequences were obtained. PDR was assessed using the WHO HIVDR surveillance mutation list.

**Results:**

Belize showed the highest PDR prevalence (17.9%), followed by Nicaragua (12.7%), Panama (12.6%), Honduras (11.7%), Mexico (8.5%, *p*<0.001) and Guatemala (7.2%, *p*<0.0001). NNRTI PDR was higher than NRTI and PI in Guatemala (4.4%, 1.7% and 1.3%, respectively), Panama (9.7%, 4.3%, 0.7%), Honduras (8.1%, 2.1%, 2.1%) and Belize (16.2%, 1.7%, 0.9%) (*p*<0.01). Belize showed higher NNRTI PDR than Nicaragua, Honduras, Guatemala and Mexico (*p*<0.01). Nicaragua showed higher NRTI PDR than Honduras and Guatemala (*p*<0.01). In all countries, clusters of drug-resistant viruses were observed with distinct geographic distribution and HIV risk factor. Comparing individuals at early chronic stage (>500 CD4+ T cells/µL) vs late chronic stage (<350 CD4+ T cells/µL), PDR in Mexico was 9.7% vs 6.5% (*p*<0.05), in Guatemala 9.5% vs 6.5% (*p*<0.05), in Honduras 14.8% vs 11.4% (*p*>0.05).

**Conclusions:**

PDR prevalence in Mexico, Guatemala, Panama, Nicaragua and Honduras remains at the intermediate level but is high in Belize. Different epidemiologic scenarios can be observed in different Mesoamerican countries warranting local HIV molecular epidemiology and TDR surveillance studies.

# Sub article: Model of HIV control and prevention strategy through integration of prevention and care at the primary care level: Mexico City HIV/AIDS Programme

**DOI:** 10.7448/IAS.18.3.20120

**Published:** 2015-04-16

**Authors:** Pablo Belaunzarán-Zamudio, Andrea González-Rodríguez, Steven Díaz, Jesús Casillas-Rodríguez, Israel Macías-González, Ubaldo Ramos-Alamillo

**Affiliations:** 1 Programa de VIH/SIDA de la Ciudad de México, Coordinación de Investigación, Mexico City, Mexico; 2 Programa de VIH/SIDA de la Ciudad de México, Dirección, Mexico City, Mexico; 3 Clínica Especializada Condesa, Dirección Médica, Mexico City, Mexico; 4 Clínica Especializada Condesa, Consejería, Mexico City, Mexico

## Abstract

**Introduction:**

We describe a model of HIV/AIDS control and prevention programme in an urban context developed for Mexico City.

**Methods:**

The programme started implementation in 2008 with the aim of increasing the access to Voluntary Counselling and Testing (VCT) services, reducing the time between HIV diagnosis, incorporation to clinical services and antiretroviral therapies (ART) initiation, and improving the effectiveness of the ART universal access programme. The main components of the model include: 1) articulation between VCT services, specialized laboratory, and provision of clinical services; 2) co-ordination between federal and local government stakeholders with civil society organizations for people living with HIV (PLWH), vulnerable populations and human rights defence; 3) outreach programmes for vulnerable populations; and 4) strengthening of clinical services provision. These services are located or centrally co-ordinated at the Clínica Especializada Condesa, a primary care clinic for HIV care.

**Results:**

Mexico City HIV/AIDS Programme integrated the provision of preventive and care services in a primary care HIV/AIDS Clinic in 2008. Annual number of HIV tests increased from 2691 to 29,799 in 2007–2014. Time between pre-VCT session and delivery of results in post-VCT session decreased from 14 days to 36 hours (median). The number of patients receiving care at Clínica Especializada Condesa increased from 3870 to 10,064 in 2008–2014. The time from access to care to ART initiation decreased from four months to two weeks. The median time to achieve undetectable HIV-VL after ART initiation decreased from 8.2 to 3.3 months in 2008 to 2012. The programme now provides services for inmates, male and female sex street-workers, people living in the street, drug users and juvenile detainees and also provides specialized clinical care for trans-gender women, acutely HIV-positive patients, STDs, and reproductive and sexual health services for teenagers and sexual violence victims.

**Conclusions:**

Mexico City HIV/AIDS Programme has implemented a multi-disciplinary approach for the prevention and control of the HIV-epidemic in an urban context in a middle-income country. The continuum between preventive, clinical care and supportive services and the cooperation of federal and local government instances with civil society organizations have been paramount for the programme's success.

# Sub article: CD4 and HIV-VL monitoring practices and costs in patients on ART in Latin America and the Caribbean

**DOI:** 10.7448/IAS.18.3.20131

**Published:** 2015-04-16

**Authors:** Pablo Belaunzarán-Zamudio, Yanink Caro-Vega, Brenda Crabtree Ramírez, Paula Luz, Beatriz Grinsztejn, Carina César, Claudia Cortés, Jean William Pape, Fernando Mejía-Cordero, Denis Padgett, Juan Sierra-Madero, Catherine McGowan, Bryan Shepherd

**Affiliations:** 1 Infectious Diseases, Instituto Nacional de Ciencias Médicas y Nutrición, Mexico City, Mexico; 2 STD/AIDS Clinical Research Laboratory, Instituto de Pesquisa Clinica Evandro Chagas/Fundacao Oswaldo Cruz, Rio de Janeiro, Brazil; 3 Área de Investigaciones Clínicas, Fundación Huésped, Buenos Aires, Argentina; 4 Fundación Arriarán, Universidad de Chile, Santiago, Chile; 5 Les Centres GHESKIO, Le Groupe Haitien d'Etude du Sarcome de Kaposi et des Infections Opportunistes, Port- au-Prince, Haiti; 6 Instituto de Medicina Tropical Alexander von Humbo, Universidad Peruana Cayetano Heredia, Lima, Peru; 7 Instituto Hondureño de Seguro Social and Hospital Escuela, Tegucigalpa, Honduras; 8 Department of Medicine, Vanderbilt University School of Medicine, Nashville, TN, USA; 9 Department of Biostatistics, Vanderbilt University School of Medicine, Nashville, TN, USA

## Abstract

**Introduction:**

We aimed to describe the frequency of CD4 and VL monitoring for HIV-positive patients (HIV + ) receiving antiretroviral therapy (ART) in Latin America and the Caribbean, assess adherence to recommendations in clinical guidelines, identify factors associated with lower frequency of monitoring and describe costs of monitoring patients on ART.

**Methods:**

We used retrospective cohort data from all CCASAnet sites during 2000–2012. Patients initiating ART were included. Follow-up time after ART initiation was divided into 180-day periods. The primary outcome was the proportion of 180-day periods with adequate monitoring, defined as having at least one CD4 and one VL measurement. In secondary analyses, adequate monitoring was based only on CD4. Factors associated with monitoring frequency were assessed using multivariable Poisson regression models, with the number of 180-day periods with adequate monitoring as outcome. Median cost for CD4 and VL measurements per patient/year were estimated.

**Results:**

A total of 14,476 patients were included. Median follow-up time was 50.4 months (IQR: 27–82.3). CD4 were monitored from a median of 2.6 measurements/year for CMH-Argentina to 1.0/year for GHESKIO-Haiti; VL measurements ranged from 2.6/year for CMH-Argentina and INCMNSZ-Mexico to 0.9/year for IHSS/HE-Honduras; VL was not regularly measured at GHESKIO-Haiti. The mean proportion of periods with adequate CD4 and VL monitoring was 61.7% (95% CI: 52.3–72.8), ranging from 85.6% at INCMNSZ-Mexico to 25.6% at IHSS/HE-Honduras. The mean proportion of 180-day periods with adequate CD4 monitoring alone was 68.6% (95% CI: 57.5–81.8%). Rates ranged from 86.5% at INCMNSZ-Mexico to 48.2% at GHESKIO-Haiti. We will show in figures in the presented data the factors associated with adequate monitoring expressed in OR: increased age, and more recent initiation of ART, which were consistently positively associated with adequate monitoring. The costs of monitoring per patient/year were US$38 for CD4 and US$140 for VL but varied substantially across the region.

**Conclusions:**

We observed that the adherence to recommendations of CD4 and VL monitoring for patients receiving ART is heterogeneous across the centres in CCASAnet, but on average is low. Costs of laboratory tests were also highly variable across sites. Our results are of use to national and international public health and policy organizations involved with HIV guideline development and implementation, and ART program cost planning.

# Sub article: P001 Utility of mobile communication devices as a tool to improve adherence to antiretroviral treatment in HIV-infected children and adolescents in Argentina

**DOI:** 10.7448/IAS.18.3.20132

**Published:** 2015-04-16

**Authors:** Erica Stankievich, Irene Foradori, Adriana Malanca, Silvina Ivalo, Marcelo Losso

**Affiliations:** Inmunocomprometidos, Hospital General de Agudos JM Ramos Mejia, Buenos Aires, Argentina

## Abstract

**Introduction:**

Optimal adherence is critical to achieve the benefits of the antiretroviral (ARV) treatment and minimizes the risk of ARV resistance. Multiple aspects are involved in adherence in children and adolescents. Although, published evidence about strategies to improve it is scarce in our setting [1–3]. The aim of this study is to evaluate the effects on adherence to ARV treatment using mobile devices as a communication strategy to improve it.

**Materials and methods:**

A prospective study was conducted in a cohort of HIV infected patients less than 25 years old. Patients taking ARV were evaluated to establish suboptimal adherence (SOA). Inclusion criteria were: HIV infection, taking ARV, viral load (VL) >1000 copies/ml, SOA reported by the primary physician, use of a mobile device. The intervention was based on mobile generic contact twice a month through any of the applications the patient chose (WhatsApp, Facebook, text message, etc.) during an eight month period. If the patient or parent required additional information, a feedback phone contact was generated. VL was performed before and after the intervention as an outcome measure of adherence.

**Results:**

Twenty-five of forty-seven patients identified as SOA were able to be contacted. One refused to participate and two have no mobile. Twenty-two patients were enrolled. Median age was 17.2 years old (range: 6–25); 15 (68%) were female; median baseline VL was 25,100 copies/ml (range: 500,000–1020 copies/ml), median log was 4.3 log (range: 3–5.7 log). Seven of twenty-two were contacted through their parents. Ten (45%) preferred to be contacted by WhatsApp, 8 (36%) by text message, 4 (18%) by Facebook and others. Each participant received a total of 16 contacts, 84% (296) were answered by the patient. Sixty-five percent (189) of the contacts generated additional requests about medications, appointments or symptoms. After eight months of strategy implementation 20/22 VL results were available. Thirteen of twenty (65%) were undetectable, 14/20 (70%) had VL <1000 copies/ml. Six of twenty (30%) VL had no changes.

**Conclusions:**

The use of mobile technology improved adherence to treatment evaluated through VL measurement. The strategy is feasible in our setting. The reminder messages trigger additional contacts between patients and provider and may lead to better engagement with HIV care. Longer follow up time is needed to evaluate the effects of this intervention in the long term.

## References

[1] Shet A, Arumugam K, Rodrigues R, Rajagopalan N, Shubha K, Raj T (2010). Designing a mobile phone-based intervention to promote adherence to antiretroviral therapy in South India. AIDS Behav.

[2] Wu AW, Snyder CF, Huang IC, Skolasky R, McGruder HF, Celano SA (2006). A randomized trial of the impact of a programmable medication reminder device on quality of life in patients with AIDS. AIDS Patient Care STDs.

[3] Simoni JM, Huh D, Frick PA, Pearson CR, Andrasik MP, Dunbar PJ (2009). Peer support and pager messaging to promote antiretroviral modifying therapy in Seattle: a randomized controlled trial. J Acquir Immune Defic Syndr.

# Sub article: P002 Clinical manifestations of Chikungunya virus infection in HIV-infected individuals: experiences during the Dominican Republic's 2014 outbreak

**DOI:** 10.7448/IAS.18.3.20133

**Published:** 2015-04-16

**Authors:** Robert Paulino, Natalia Gomez, Paola Lockhart, Ellen Koenig, Adrian Puello

**Affiliations:** 1 Research Department, School of Medicine, Universidad Iberoamericana, Santo Domingo, Dominican Republic; 2 Medical Department, Instituto Dominicano de Estudios Virologicos, Santo Domingo, Dominican Republic; 3 Epidemiology, Hospital General Plaza de la Salud, Santo Domingo, Dominican Republic

## Abstract

**Introduction:**

Chikungunya virus (CHIKV) is an alpha virus causing acute febrile illness characterized by crippling arthralgia [1], and a disseminated rash [2]. First recognized in 1953 in East Africa, CHIKV has been spread over the world due to the expansion of Aedes mosquitoes within warm countries [3]. CHIKV is an emerging disease in The Americas after the first cases were recognized in the lesser islands of the Caribbean [4] known as the Lesser Antilles. Immune modulatory response to CHIKV infection appears to be T lymphocyte and macrophage-mediated [5], which is the same for long-lasting chronic arthralgia in immunosuppressed and immune deprived individuals [4]. People living with HIV are included among the high-risk populations for atypical clinical manifestations of CHIKV infection [1]. The purpose of this study was to determine the clinical findings related to CHIKV infection in two groups in the Dominican Republic based on their immune status.

**Materials and methods:**

During the most recent outbreak of CHIKV in the Caribbean, we selected randomly from two outpatients centres in the capital city Santo Domingo, Dominican Republic. The sample consisted of 100 participants meeting the CDC case definition for CHIKV infection. Random distribution of cases (HIV negative) and controls (HIV positive) were allocated based on HIV known status at the moment of presenting to care. After informed consent IgM antibodies were identified to differentiate dengue from CHIKV infections. CD4+ T lymphocytes and HIV viral load, and HIV medications were reviewed. Epidemiological data was analyzed with Epi Info™7.

**Results:**

A total of 100 participants fulfilled inclusion criteria. Distribution of cases and controls was 1:1. Men constituted 23% (*n*=23) of total sample. The mean age for men was 44.04 years (SD=0.5), and for women 45.29 years (SD=1.1). Immune status of HIV-positive participants assessed by CD4+ T lymphocytes revealed that 76.4% (*n*=39) had a CD4+ T levels greater to 350 cells/µl, and 74.5% (*n*=38) with HIV viral load <40 copies/ml. A similar distribution of clinical findings was observed despite immune status, with a clinically significant difference in fever and lower limb arthralgia, which were more prevalent in HIV negative. Only nausea was significantly higher in HIV-positive individuals (Table 1).

**Conclusions:**

This study comparing clinical manifestations in HIV-negative and -positive patients during the acute phase of Chikungunya did not reveal any major differences in acute phase symptoms. The effect of lower CD4+ T cells on acute symptoms must be studied in patients with more advanced disease.

**Abstract P002–Table 1:** Clinical findings of CHIKV infection in HIV-positive and HIV-negative cohorts

Signs and symptoms	VIH (+) (%)	% (95% CI)	VIH (–) (%)	% (95% CI)	Odds (*p*)
Fever	48 (94.11)	(87.65–100%)	49 (100)	(100–100%)	0.1400 (*p*=0.1974)
Headache	35 (68.62)	(55.88–81.36%)	37 (75.51)	(63.47–87.55%)	0.7095 (*p*=0.4444)
Myalgia	48 (94.11)	(87.65–100%)	46 (93.87)	(87.15–100.59%)	1.0435 (*p*=0.9597)
Upper limb arthralgia	47 (92.15)	(84.77–99.53%)	47 (95.91)	(90.36–101.46%)	0.6667 (*p*=0.6648)
Lower limb arthralgia	47 (92.15)	(84.77–99.53%)	49 (100)	(100–100%)	0.1371 (*p*=0.1927)
Arthritis	36 (70.58)	(58.07–83.09%)	38 (77.55)	(65.87–89.23%)	0.6947 (*p*=0.4286)
Rash	37 (72.54)	(60.29–84.79%)	35 (71.42)	(58.77–84.07%)	0.9600 (*p*=0.9262)
Nauseas	15 (29.41)	(16.91–41.91%)	24 (48.97)	(34.97–62.97%)	0.4340 (*p*=0.0467)
Vomiting	9 (17.64)	(7.18–28.1%)	12 (24.48)	(12.44–36.52%)	0.6607 (*p*=0.4027)
Diarrhoea	17 (33.33)	(20.39–46.27%)	17 (34.69)	(21.36–48.02%)	0.9412 (*p*=0.8858)
Abdominal pain	21 (41.17)	(27.66–54.68%)	23 (46.93)	(32.96–60.9%)	0.7913 (*p*=0.5619)
Others (pneumonia, encephalitis, Guillain-Barre)	16 (31.38)	(18.64–44.12%)	29 (59.18)	(45.42–72.94%)	0.3153 (*p*=0.0059)

## References

[1] Hassan R, Rahman M, Moniruzzaman M, Rahim A, Barua S, Biswas R (2014). Chikungunya – an emerging infection in Bangladesh. J Med Case Rep.

[2] Thiberville SD, Boisson V, Gaudart J, Simon F, Flahault A, de Lamballerie X (2013). Chikungunya fever. PLoS Negl Trop Dis.

[3] Wiwanitkit S, Wiwanitkit V (2013). Ckikungunya virus infection and relationship to rainfall, the relationship study from southern Thailand. J Arthropod Borne Dis.

[4] Centers for Disease Control and Prevention http://wwwnc.cdc.gov/travel/notices/watch/chikungunya-saint-martin.

[5] Teo T, Lum FM, Claser C, Lulla V, Lulla A, Merits A (2013). A pathogenic role for CD4+ T cells during Chikungunya virus infection in mice. J Immunol.

# Sub article: P003 Specific neutralizing antibody detection against chikungunya virus in HIV-positive and HIV-negative individuals, Dominican Republic 2014

**DOI:** 10.7448/IAS.18.3.20134

**Published:** 2015-04-16

**Authors:** Robert Paulino, Paola Lockhart, Natalia Gomez, Adrian Puello

**Affiliations:** 1 Research Department, School of Medicine, Universidad Iberoamericana, Santo Domingo, Dominican Republic; 2 School of Medicine, Universidad Iberoamericana, Santo Domingo, Dominican Republic; 3 Epidemiology, Hospital General Plaza de la Salud, Santo Domingo, Dominican Republic

## Abstract

**Introduction:**

By April 2013, the first autochthonous transmission of chikungunya virus (CHIKV) in the Dominican Republic was confirmed [1]. A total of 537,628 suspected cases were recorded by EW51 in 2014, and 84 confirmed cases [2]. According to the PAHO/WHO a confirmed case is a suspected case with any specific CHIK test (viral isolation, RT-PCR, IgM or four-fold increase of CHIKV specific antibodies titters) [3]. The objective of this study was to measure the presence of IgM-specific neutralizing antibodies among HIV-positive individuals after clinical onset of symptoms during the last outbreak in the Dominican Republic.

**Materials and methods:**

During the peak of incidence of cases, patients attending an outpatient clinic for HIV-positive individuals in Santo Domingo with signs and symptoms suggestive of CHIKV infection (fever>38°C, severe arthralgia or arthritis) were asked to participate in this study. After informed consent was collected a blood sample was obtained for CHIKV-IgM detection and analyzed using reagents provided by Aria CHIKV IgM detection (CTK Biotech, CA).

**Results:**

A total of 100 cases were tested. Women represented 73% (*n*=37), while men represented 27% (*n*=14). The immunological status in HIV positive was measured with the level of CD4+ T cells at the moment of onset of symptoms; 76.4% (*n*=39) having greater than 350 cells/µl, followed by 19.6% (*n*=10) with 101–350 cells/µl. HIV viral load was undetected in 74.5% (*n*=38) of the sample, and 11.7% (*n*=6) with a VL >50,000 copies/ml. CHIKV-IgM detection in HIV(+) was positive in 21.57% (*n*=11), and 30.61% in HIV(–). First positive IgM detection in HIV(–) was observed after one month of onset of symptoms; while in HIV(+) was 2w 1d (Figure 1 and Table 1).

**Conclusions:**

This study suggests that immunological status is not affecting earlier inflammatory markers against CHIKV infection in HIV(+) individuals. Noteworthy, persistence of specific CHIKV IgM was detected after four months of onset of symptoms in HIV(+). Comparison of CHIKV-IgM in acute onset with an immune competent cohort demonstrates that rapid diagnostic test may be useful among immunosuppressed individuals to differentiate Dengue virus infection and CHIKV in the Americas where both pathogens co-circulate due to the presence of competent Aedes vectors.

**Abstract P003–Figure 1:** CHIKV specific IgM detection in HIV (−) and HIV (+) individuals after onset of symptoms, Dominican Republic 2014.

**Abstract P003–Table 1:** CHIKV IgM detection in HIV (+) and HIV (–) individuals, Dominican Republic 2014

	HIV(+) *n*=51		HIV (–) *n*=49		
	(*n*)	(%)	(*n*)	(%)
CHIKV IgM (+)	11	21.57	15	30.61
CHIKV IgM (–)	40	78.43	34	69.39

## References

[1] PRO/EDR> Chikungunya (25): Caribbean. http://promedmail.chip.org/pipermail/promed/2014-April/003851.html.

[2] PAHO/WHO Chikungunya cumulative cases in the Americas, EW1 2015 [Internet]. http://www.paho.org/hq/index.php?Itemid=40931.

[3] PAHO (2011). Preparedness and Response for Chikungunya Virus: Introduction in the Americas. http://www.paho.org/hq/index.php?option=com_topics&view=article&id=343&Itemid=40931&lang=en.

# Sub article: P004 HIV treatment cascade in HIV incarcerated patients in Mexico City 2014

**DOI:** 10.7448/IAS.18.3.20135

**Published:** 2015-04-16

**Authors:** Hugo Vargas González, Jesús Casillas-Rodríguez, Nathalie Gras Allain

**Affiliations:** 1 Consulta Externa, Clínica Especializada Condesa, Mexico City, Mexico; 2 Dirección Médica, Clínica Especializada Condesa,, Mexico City, Mexico; 3 Subdirección Vínculación, Clínica Especializada Condesa, Mexico City, Mexico

## Abstract

**Introduction:**

In 2011, Centers for Disease Control and Prevention (CDC) published the first HIV treatment cascade with data from 2008 and sets the basis to implement health policy in the areas of opportunity to stop the epidemic [1]. Approximately 40,000 people are interned in the prison system in Mexico City, and the estimated HIV prevalence is 1%. In order to improve survival rates, quality of life and reduce the transmission of the infection, we have to look at the timely diagnosis, linkage and retention of patients in medical care and obviously prevention and treatment (highly active antiretroviral therapy (HAART)). Incarcerated persons with HIV have particular characteristics that define them: overcrowding, multiple sex partners, drug use, psychiatric conditions, antisocial personalities, low education levels, etc. Having HIV patients concentrated in the same place with supervised ARV daily dosage intake results in better adherence, retention in care and thus better virology control decreasing infection probability.

**Materials and methods:**

It is a retrospective study. We used data from the National System for Logistics Administration and Surveillance of ARV in Mexico (SALVAR in Spanish) up to the 31 December 2014, the database of the CIENI/CENSIDA and the database of the HIV Prison Programme in Mexico City. Criteria for inclusion: Incarcerated male patients with HIV infection in Mexico City during 2014 in Santa Martha Acatitla Penitentiary.

**Results:**

We started 2014 with 184 patients, adding 60 new patients throughout the year, 13 recidivists, 67 were freed and 9 died. At 31 December 2014, 206 HIV patients remained incarcerated, of which 92.2% (190) are linked and retained to health care (concentrated in the prison of Santa Martha Acatitla), of which 87.4% are on HAART, with 72.8% under virology control (VL<200) and 63.1% undetectable (VL<40) (Figure 1). Twenty-one percent (60) initiated HAART in the previous six months, 5.2% (10) initiated their HAART protocol, in virology failure 2.1% (4), 1% (2) with persistent low grade viraemia (VL<1000) and 2.1% (4) with blip. Of the 67 patients that were freed, 91% (61) continued their medical treatment at the Condesa Specialized Clinic.

**Conclusions:**

It is necessary to strengthen the diagnosis of HIV in prison settings. Linkage and retention in medical care is covered in this model (HIV Programme in Prisons), alongside working with improving adherence to HAART in order to increase the levels of undetectability. The model of supervised daily dosage has given partial effective results given that the ARV is provided daily but it does not guarantee that the patients swallow the pills. Another important problem is how to link 100% of patients to ambulatory care once they obtain their freedom. The HIV Programme in Prisons of Mexico City is effective and can be replicated in different penitentiary systems in other states and countries, but not in the general population.

**Abstract P004–Figure 1:** Data from the National System for Logistics Administration and Surveillance of ARV in Mexico (SALVAR) 31 of December, 2014. Database of the CIENI/CENSIDA, 2014. Database of the HIV Prison Programme in Mexico City 2014.

## References

[1] Cohen SM, Van Handel MM, Branson BM, Blair JM, Hall HI, Hu X (2011). Vital signs: HIV prevention through care and treatment–United States. MMWR Morb Mortal Wkly Rep.

# Sub article: P005 Impact of conditional economic incentives to reduce risky behaviours among high-risk men who have sex with men in a three-year randomized pilot study

**DOI:** 10.7448/IAS.18.3.20136

**Published:** 2015-04-16

**Authors:** Sandra Sosa-Rubi, Omar Galarraga, Don Operario, Biani Saavedra, Kenneth Mayer, Nathalie Gras Allain, Carlos J Conde-Gonzalez, Andrea Gonzalez

**Affiliations:** 1 Health Economics Department, Instituto Nacional de Salud Pública, Cuernavaca, Morelos, Mexico; 2 Brown University, Providence, RI, USA; 3 Clinica Condesa, Mexico City, Mexico; 4 Department of Microbiology, Instituto Nacional de Salud Pública, Cuernavaca, Mexico

## Abstract

**Introduction:**

We test conditional economic incentives (CEI) to motivate self-protection and health-care-seeking behaviours among male sex workers (MSW) in Mexico City. We present baseline and follow-up results for MSW, aged 18–30, recruited in Mexico City (*n*=227).

**Materials and methods:**

Participants were randomized into four groups. In Treatment 1 (*n*=57) participants received a medium CEI (~$50 USD/each time) only if they were free of new, curable STIs at months 6 and 12. Treatment 2 (*n*=53) received a high CEI (~$75 USD/each time) only if they were free of new, curable STIs at months 6 and 12. Controls (*n*=61) did not receive any incentives even if they were free of STIs at months 6 and 12. Participants in the unconditional economic incentive (UEI) arm (*n*=56) received a medium incentive (~$50 USD at months 6 and 12) regardless of STI status. In addition, everyone received inconvenience fees (~$10 USD/each time at baseline, month 6 and month 12). We recruited from various sites in Mexico City. All eligible men took part in a standard one-hour HIV education/information session after completing baseline measures, and before random assignment. All received HIV/STI testing; those infected were offered treatment (for curable STI). Chronic STIs were not used for the conditional incentives. Any participant that was HIV+ was allowed to continue in the trial and was referred to treatment as indicated by Mexico guidelines. Unadjusted and adjusted models were estimated with random effects and robust standard errors; and interactions that can modify the effect of the incentives.

**Results:**

Higher incentives were associated with higher participation rates. Incidence of syphilis was lower (AOR: 0.4; CI: 0.1–1.0) among participants in the high-CEI group compared to the control group. Greater condom use self-efficacy was found among participants of the UEI group (AOR: 5.4; CI: 1.1–26.6) compared to the control group; and significant reduction in the number of non-commercial sexual partners was found in the medium-CEI group (coefficient: −1.6; CI: −3.3–0.0).

**Conclusions:**

CEI and UEI are feasible and acceptable among MSW in Mexico City. CEI and UEI seem to help to reduce risk behaviours. Inconvenience fees (transport compensation) help increase retention and linkage to care. A larger randomized controlled trial is justified to test CEI and UEI using a fully-powered sample size for primary and secondary outcomes.

# Sub article: P006 HIV rapid testing in an emergency room at a low prevalence setting

**DOI:** 10.7448/IAS.18.3.20137

**Published:** 2015-04-16

**Authors:** Juan Luis Mosqueda, Alejandro Macias Hernandez, Monica Lopez Segura

**Affiliations:** 1 Secretary Health, HIV Clinic, Leon, Mexico; 2 Department of Medicine, University of Guanajuato, Leon, Mexico

## Abstract

**Introduction:**

Early diagnosis of HIV infection is crucial for early treatment, which can lead to better individual prognosis and some benefits in public health, such as reduction of the HIV transmission. One of the proposed strategies to improve the opportunity of early diagnosis is to widespread the HIV screening to different settings, including health care units. It has been shown that routine HIV screening could be justified in cost-effectiveness terms in settings where the prevalence of undiagnosed HIV is 0.1% or greater. The aim of the present study was to determine the prevalence of undiagnosed HIV infection at the emergency room in a city considered to have a low prevalence of HIV in its population [1–5].

**Materials and methods:**

A cross-sectional study was performed at the Hospital General Regional de Leon (HGRL). Leon is the biggest city in Guanajuato, which is located at the centre in Mexico and is considered one of the states with lowest prevalence of HIV in the country. During a 10-month period, HIV rapid testing was offered to all patients attended in the emergency room area, counselling was given to all patients and those who accepted to participate signed an informed consent. Patients were excluded from HIV testing if they (1) were unable to provide consent for HIV testing as determined by the clinical staff (e.g. altered mental status or critical illness); (2) were detainees or prisoners; or (3) self-identified as being infected with HIV. Rapid HIV test was made using the NEOGEN^®^ test. If the first test was negative no further tests were made, and the result was reported as non-reactive. If the first test was positive, confirmation was made by RT-PCR. Results were analyzed and presented using descriptive statistics.

**Results:**

During the study period 1823 rapid tests were made, 17 of them were reactive and 15 were confirmed as positive by RT-PCR, for a prevalence of undiagnosed HIV infection of 0.8% (95% CI: 0.4–1.2). Acceptance rate was 96%.

**Conclusion:**

Considering the high acceptance for HIV rapid testing and the prevalence of undiagnosed HIV infection found, we can conclude that widespread screening of HIV in health care settings should be considered even in regions where the assumed prevalence is low.

## References

[1] Sanders GD, Bayoumi AM, Sundaram V, Pinar Bilir S, Neukermans CP, Rydzaket CE (2005). Cost-effectiveness of screening for HIV in the era of highly active antiretroviral therapy. N Engl J Med.

[2] Haukoos JS, Hopkins E, Byyny RL, Denver Emergency Department HIV Testing Study Group (2008). Patient acceptance of rapid HIV testing practices in an urban emergency department: assessment of the 2006 CDC recommendations for HIV screening in health care settings. Ann Emerg Med.

[3] Haukoos JS, Hopkins E, Byyny RL, Conroy AA, Silverman M, Eisert S (2009). Design and implementation of a controlled clinical trial to evaluate the effectiveness and efficiency of routine opt-out rapid human immunodeficiency virus screening in the emergency department. Acad Emerg Med.

[4] Burns F, Edwards SG, Woods J, Haidari G, Calderon Y, Leider J (2013). Acceptability, feasibility of costs of universal offer of rapid point of care testing for HIV in an Acute admissions unit: results of the RAPID Project. HIV Med.

[5] Lyons MS, Lindsell CJ, Wayne DB, Ruffner AH, Hart KW, Fichtenbaum CJ (2011). Comparison of missed opportunities for earlier HIV Diagnosis in 3 geographically proximate emergency departments. Ann Emerg Med.

# Sub article: P007 Gender approach: violence in women and transgender women with HIV in clinic in Mexico City

**DOI:** 10.7448/IAS.18.3.20138

**Published:** 2015-04-16

**Authors:** Harumi Hirata, Hamid Vega-Ramirez, Jeremy Cruz, Victor Rodriguez, Daniela Ferreyra, Carolina Rocabert

**Affiliations:** Mental Health Program, Condesa Specialized Clinic, Mexico, Mexico

## Abstract

**Introduction:**

Violence and HIV are two problems of public health that affects millions of women worldwide [1]. At least one in three women worldwide has suffered physical, economic, sexual or psychological violence in their lifetime [2,3]. Belonging to the female gender increases the risk of receiving violence [4]. A history of violence has been associated with an increased risk for transmission/acquisition of HIV and poor medication adherence [5].

**Materials and methods:**

Of the information collected between 2013 and 2014 amongst 1773 patients recently diagnosed with HIV Condesa Specialized Clinic, 199 were women and transgender women. We asked for socio-demographic data, history of violence, the stage in life which the presented violence and perpetrators. STATAv13 was used. Frequencies and percentages of groups of women and transwomen were performed. Comparisons between the men, women and transgender were performed taking the history of violence, Chi^2^ used for the OR.

**Results:**

The 80.4% (*n*=160) were women and 19.5% (*n*=39) were transgender women. In the group of women 46% (*n*=73) were single, average age 34 years (SD±9.9), mean education 8.5 years (SD±9.9), 47.5% (*n*=76) have a paid employment of these only 0.62% (*n*=1) is engaged in sex work. In the group of transgender women 92.3% (*n*=36) were single, average age 34 years (SD±9.1), mean education 9.2 years (SD±3.9), 92.3% (*n*=36) have gainful employment of these 30.7% (*n*=12) were engaged in sex work. It was reported that 57.8% (*n*=115) have a history of violence throughout the life of the patients, 46.8% (*n*=93) were female and 11% (*n*=22) were transwomen (*X* ^2^=23.14, *p*≤0.001). The 42% (*n*=39) of women with a history of violence reported that the main perpetrator of the violence was the couple and 19.3% (*n*=18) a family being the second most common. The 27.2% (*n*=6) of women-trans reported that a relative had been the perpetrator and 18.1% (*n*=4) were family members. Women with a history of violence had OR of 1.43 (95% CI (1.04–1.96)) and the transgender women OR was 1.29 (95% CI (0.68–2.43)) with *p*≤0.001.

**Conclusions:**

The 57.8% of women and transgender women diagnosed with HIV have a history of violence throughout life. Both groups are victims of partner and family violence, implying that have poor support network. These suggest that female gender is a factor in being the recipient of violence. Women and transgender women have increased vulnerability which can influence the early detection of disease and treatment compliance.

## References

[1] Heise L, Ellsber M, Gottemoeller M (1999). Ending violence against women. Populations Reports.

[2] Connell RW (1987). Gender and power: society, the person and sexual politics.

[3] Whetten K, Leserman J, Lowe K, Stangl D, Thielman N, Swartz M (2006). Prevalence of childhood sexual abuse and physical trauma in an HIV positive sample from the deep south. Am J Public Health.

[4] Wilson SM, Sikkema KJ, Randy KW (2014). Gender modarates the influence of psychosocial factors and drug use on HAART adherence in the context of HIV and childhood sexual abuse. AIDS Care.

[5] Machtinger EL, Wilson TC, Haberer JE, Weiss DS (2012). Psychological trauma and PTSD in HIV-positive women; meta-analysis. AIDS Behav.

# Sub article: P008 HIV-1 pol genetic diversity and antiretroviral resistance mutations in illicit drug users from Piauí, Northeast Brazil

**DOI:** 10.7448/IAS.18.3.20139

**Published:** 2015-04-16

**Authors:** Aldemir Oliveira-Filho, Pricila Pereira, Laine Pinto, Leila Sawada, Jairo Castro, Roseane Lima-Verde, José Alexandre Lemos, Antonio Vallinoto, Evaldo Oliveira

**Affiliations:** 1 Institute for Coastal Studies, Federal University of Pará, Bragança, Brazil; 2 Institute of Biological Sciences, Federal University of Pará, Belém, Brazil; 3 Laboratory of Molecular Biology, Center for Hematology and Hematology of Pará, Belém, Brazil; 4 Center for Health Sciences, Federal University of Piauí, Teresina, Brazil

## Abstract

**Introduction:**

Antiretroviral therapy (ART) has reduced morbidity and mortality related to human immunodeficiency virus (HIV) infection, but in spite of this advance, HIV mutations decrease antiretroviral susceptibility, thus contributing to treatment failure in patients [1]. Illicit drug users (DUs) are vulnerable to HIV and other blood-borne pathogens as a result of sharing contaminated syringes and other equipment [2]. In Piauí, there is a high prevalence of HIV-1 infection among DUs [3]. There is a paucity of information on the circulation of HIV-1 subtypes and the resistance to the primary drugs in this vulnerable group. This study described the prevalence of primary HIV-1 drug resistance and subtypes circulating in DUs from Piauí, Northeast Brazil.

**Materials and methods:**

This cross-sectional study of a non-probabilistic convenience sample was based on biological samples provided by DUs attended at Central Laboratory and STD/AIDS Reference Unit of the State of Piauí. In total, 107 DUs were recruited in Teresina, Northeast Brazil [3]. All DUs were antiretroviral naïve patients. Protease and partial reverse transcriptase regions were retrotranscribed from plasma HIV-1 RNA and were sequenced after direct nested PCR. HIV-1 subtype was assigned by phylogenetic analysis. Primary drug resistance was analyzed by the Calibrated Population Resistance (CPR) tool using Stanford Surveillance Drug Resistance Mutation (SDRM) and International AIDS Society-USA (IAS-USA) major mutation list.

**Results:**

Primary drug resistance mutations ranged from 10% (IAS-USA) to 14% (SDRM). High level resistance to at least one antiretroviral drug was observed. T215D/S revertant mutations were identified in 8/107 patients. HIV-1 subtype B represented 84.1%, subtype F1 7.5%, subtype C 3.7%, B/F1 2.8% and two samples was a F1/C/B mosaic. HIV-1 subtype C sequences formed a monophyletic cluster with other Brazilian subtype C sequences.

**Conclusions:**

Our HIV-1 pol sequences from Piuaí include the important inland HIV-1 subtype C sequences and help compose the molecular epidemiology map of HIV-1 in Brazil. This data also show that a significant proportion of DUs presented important drug resistance mutations. Therefore DUs from this setting may benefit from pre-treatment genotypic testing to optimize the choice of antiretroviral drugs and to help control HIV-1 transmission.

## References

[1] Little SJ, Frost SD, Wong JK, Smith DM, Pond SL, Ignacio CC (2008). Persistence of transmitted drug resistance among subjects with primary human immunodeficiency virus infection. J Virol.

[2] Degenhardt L, Hall W (2012). Extent of illicit drug use and dependence, and their contribution to the global burden of disease. Lancet.

[3] Oliveira E, Silva L, Lima-Verde R, Aragao AL, Castro J, Sawada L (2014). Epidemiological aspects of HIV infection among illicit drug users in the State of Piauí, Northeast Brazil. J Int AIDS Soc.

# Sub article: P009 Evaluation of HIV-infection control, risk behaviours and comorbidities among inmates in Mexico

**DOI:** 10.7448/IAS.18.3.20140

**Published:** 2015-04-16

**Authors:** Jaime Andrade-Villanueva, Luz González-Hernández, Mara Llamas-Covarrubias, David Ponce-Herrera, Raul Soria-Rodríguez, Sergio Zuñiga-Quiñonez, Pedro Martínez-Ayala

**Affiliations:** 1 HIV Unit, Universidad de Guadalajara, Guadalajara, Mexico; 2 HIV Unit, Hospital Civil de Guadalajara, Guadalajara, Mexico

## Abstract

**Introduction:**

A high prevalence of HIV infection in prisons has been recognized as a significant health problem in developed countries. To this point, it is important to evaluate our prison health-system to see if the model of care is adequate or not and to look for new care strategies to achieve good HIV-control into prisons.

**Materials and methods:**

Cross-sectional, retrospective study of HIV-positive prisoners. To describe the main risk behaviours for HIV-infection, its control, the prevalence of comorbidities and other co-infections in prisoners who were treated in the HIV-Unit of Hospital Civil de Guadalajara, Mexico. All HIV-infected prisoners who were receiving medical care at our HIV-unit from May 1st 2013 to May 31st 2014 were included. Data were obtained from an electronic database of medical records.

**Results:**

A total of 88 patients were included, 95% were male, with a median age of 36 years, all Hispanic. The principal risk factors to HIV-infection were unprotected heterosexual intercourse (55%), following of homo/bisexual intercourse, intravenous drug use (45 and 33%, respectively). The 71% of patients were in an advanced stage of HIV infection and 72% of the patients on HIV-treatment, reached HIV control with undetectable viral load. The 21% had Syphilis. We found a serologic evidence of Hepatitis C co-infection in 33%, the majority was genotype 1a and none received HCV treatment, moreover, the 8% of patients had serologic markers for hepatitis B. Regarding opportunistic infections; pulmonary tuberculosis was the most frequent (36%), followed by disseminated histoplasmosis (10%). The principal comorbidity was dyslipidaemia in 42 and 16% had metabolic syndrome. The 89% used to smoke daily, 76% used any kind of drug (principally, marijuana and cocaine) and 85% were alcohol consumers.

**Conclusions:**

Efficiency of highly active antiretroviral therapy (HAART) among our prisons is higher compared to other cohorts [1]; however, the high frequency of comorbidities, smokers and drugs users, could increase the mortality and complicate HIV-infection by itself [2,3]; moreover, the high prevalence of HCV and HBV could be indicative of potentially risky blood-borne exposures or unprotected sexual contacts. So, new care strategies are needed for integral treatment of these patients.

## References

[1] Springer SA, Spaulding AC, Meyer JP, Altice FL (2011). Public health implications for adequate transitional care for HIV-infected prisoners: five essential components. Clin Infect Dis.

[2] Jürgens R, Nowak M, Day M (2011). HIV and incarceration: prisons and detention. J Int AIDS Soc.

[3] Helleberg M, May MT, Ingle SM, Dabis F, Reiss P, Fätkenheuer G (2015). Smoking and life expectancy among HIV-infected individuals on antiretroviral therapy in Europe and North America. AIDS.

# Sub article: P010 HIV/STI prevalence and sociodemographic data in incarcerated female adolescents in Mexico City

**DOI:** 10.7448/IAS.18.3.20141

**Published:** 2015-04-16

**Authors:** Teresita de Jesús Cabrera López, Ubaldo Ramos-Alamillo, Carlos Cruz Palacios, Andrea González-Rodríguez, Elena Langarica Naves, Steven Díaz

**Affiliations:** 1 Ginecología y Obstetricia, Clinica Especializada Condesa, Mexico City, Mexico; 2 Dermatología, Clinica Especializada Condesa, Mexico City, Mexico; 3 Jefe de Programa, Clinica Especializada Condesa, Mexico City, Mexico; 4 Director de Prevención, Clinica Especializada Condesa, Mexico City, Mexico

## Abstract

**Introduction:**

Determine HIV and sexually transmitted infections (STI) prevalence and respective sociodemographic.

**Materials and methods:**

Cross-sectional retrospective study performed at Condesa Specialized Clinic of Public Health Services of the Federal District. The sample was made up of female adolescents in juvenile correctional facilities in Mexico City. All patients have a clinical history, physical exam, cervical cytology, colposcopy, lab exams for HIV, HBV, HCV and Syphilis.

**Results:**

Seventy-two patients were studied. The average age was 16.5 years. Education was: 45.8% primary, 40.2% secondary and 8.33% without schooling. The average age for onset of sexual activity was 14 years and the average number of sexual partners was 4.4. Reported frequency of condom use was 70.8%; 55.5% were nulligravid and 38.8% had been pregnant once (two adolescents were pregnant during physical examinations), 2.7% had two previous pregnancies and 2.7% has three previous pregnancies and 8.3% had at least one abortion. All adolescents that had previous pregnancies had only primary education. 11.1% reported incidents of sexual violence during childhood and 81.9% reported some form of drug use, with an average age of onset for drug use at 12 years. The drugs reported with more use were: inhalants (55.5%), alcohol (43%), tobacco (37.5%) and cannabis (31.9%). Most of the drug using adolescents only had primary education. STIs were found in 33.3% of the population, the more frequent being: acuminated condiloma and low grade intraepithelial lesions (6.9% each). In second place Molluscum Contagiosum and high grade intraepithelial lesions (5.5% each), Human Papilloma Virus and Syphilis (4.1% each). HIV was 1.3%. In the group of patients with an STI, 12.5% had two or more. The age of onset for sexual activity for the adolescents with STI was 13.8 years and had an average of about five sexual partners at the time of the medical exploration. About half of adolescents with an STI (54%) had primary education and the other half (46%) had secondary education.

**Conclusions:**

HIV prevalence in this group of adolescent females was high. Drug use and pregnancy were related to educational levels attained while early age of onset of sexual activity was more related to having an STI. Sexual education interventions are needed for adolescents in correctional facilities for further prevention of STI and unwanted pregnancies.

# Sub article: P011 91–94% SVR24 in HCV/HIV-1 co-infected patients treated with ombitasvir/paritaprevir/r and dasabuvir with ribavirin

**DOI:** 10.7448/IAS.18.3.20142

**Published:** 2015-04-16

**Authors:** Chia Wang, Roger Trinh, Peter Ruane, David Wyles, Joseph Eron, Laveeza Bhatti, Yiran Hu, Barbara McGovern, Nancy Shulman, Mark Sulkowski

**Affiliations:** 1 Infectious Diseases, HIV/AIDS Clinic, Virginia Mason Medical Center, Seattle, WA, USA; 2 AbbVie Inc., GPRD, North Chicago, IL, USA; 3 Infectious Diseases, HIV and Hepatitis, Peter J. Ruane, MD, Inc.,, Los Angeles, CA, USA; 4 Medicine, Infectious Diseases, University of California San Diego, San Diego, CA, USA; 5 Infectious Diseases, University of North Carolina, Chapel Hill, NC, USA; 6 AIDS Healthcare Foundation, Beverly Hills, CA, USA; 7 Statistics, AbbVie Inc., North Chicago, IL, USA; 8 Viral Hepatitis Center, Johns Hopkins University, Baltimore, MD, USA

## Abstract

**Introduction:**

New interferon-free treatments for hepatitis C virus (HCV) infection have demonstrated response rates in HCV/HIV-1 co-infected patients similar to those observed in large phase three studies conducted in HCV monoinfected populations. The 3 direct-acting antiviral (3D) regimen of ombitasvir, paritaprevir (identified by AbbVie and Enanta; co-dosed with ritonavir), and dasabuvir with ribavirin (RBV) achieves high sustained virologic response (SVR) rates in HCV genotype (GT) 1-monoinfected patients. SVR 12 rate was 91–94% with the 3D+RBV regimen in adults with HCV GT1 and HIV-1 co-infection with and without cirrhosis.

**Methods:**

Part 1a of the TURQUOISE-I study is a randomized, open-label assessment of the 3D+RBV regimen for 12 or 24 weeks in HCV treatment-naïve or pegIFN/RBV-experienced patients, with or without cirrhosis. Eligible patients must have had CD4+ count ≥200 cells/mm^3^ or CD4+% ≥14%, and plasma HIV-1 RNA suppressed on a stable atazanavir- or raltegravir-inclusive antiretroviral (ART) regimen. Intent-to-treat (ITT) results for SVR at post-treatment week 24 (SVR24) and 95% confidence intervals are presented.

**Results:**

Of 63 patients enrolled, 92% were male, 24% were black race, 19% had compensated cirrhosis and 16% had a prior null response to pegIFN/RBV treatment. After 3D+RBV treatment for 12 or 24 weeks, 94 and 91% of patients achieved SVR24, respectively (Table 1); no patients experienced virologic relapse between post-treatment week 12 and 24. Two patients in the 24-week treatment group had evidence of HCV re-infection by nucleotide sequence and phylogenetic analyses. The majority of adverse events (AEs) were mild or moderate, and no serious AE or discontinuations due to an AE were reported. The most common AEs were fatigue (48%), insomnia (19%) and nausea (18%). Elevation in total bilirubin was the most common laboratory abnormality, occurring predominantly in patients receiving atazanavir. No patient had a confirmed HIV-1 breakthrough ≥200 copies/ml during treatment.

**Conclusions:**

In treatment-naïve and -experienced GT1 HCV/HIV-1 co-infected patients with or without cirrhosis, the high rates of virologic response and low rate of treatment discontinuation were consistent with those in HCV GT1-monoinfected populations receiving 3D+RBV.

**Abstract P011–Table 1:** Virologic response rates during and after treatment

	12-week 3D + ribavirin (*N*=31)	24-week 3D + ribavirin (*N*=32)
Response, *n* (%) [95% CI]		
RVR	31 (100) [89–100]	32 (100) [89–100]
EOTR	30 (97) [84–99]	31 (97) [84–100]
SVR12	29 (94) [79–98]	29 (91) [76–97]^a^,^b^
SVR24	29 (94) [79–98]	29 (91) [76–97]^a^,^b^
Reasons for not achieving SVR12, *n* (%)		
Early study discontinuation	1 (3)	0
Virologic breakthrough during treatment	0	1 (3)
Virologic relapse after treatment	1 (3)	0
HCV re-infection after treatment	0	2 (6)^a^,^b^

a Denotes one patient with HCV re-infection at post-treatment week 8

b denotes one patient with HCV re-infection at post-treatment week 12.

Ninety-five percent confidence intervals calculated using the Wilson score method for the binomial distribution.

3D, ombitasvir/paritaprevir/r and dasabuvir; RVR, rapid virologic response; EOTR, end of treatment response; SVR12, sustained virologic response at post-treatment week 12; SVR24, sustained virologic response at post-treatment week 24.

# Sub article: P012 Genotypification of Human Papilloma Virus in women that live with the Human Immunodeficiency Virus in a specialized clinic in Mexico City

**DOI:** 10.7448/IAS.18.3.20143

**Published:** 2015-04-16

**Authors:** Robert Antonio Duen̈as Dominguez, Ubaldo Ramos-Alamillo, Veronica Ruiz

**Affiliations:** Servicios De Salud Del Distrito Federal, Clinica Especializada Condesa, Mexico City, Mexico

## Abstract

**Introduction:**

The infection of the human papilloma virus (HPV) of high risk (HR) is a necessary factor for cervical lesions and invasive cervical cancer (ICC) [1]. In women with human immunodeficiency virus (HIV), HPV infections are more frequent with a higher risk of ICC [2,3].

**Materials and methods:**

Learn the HPV (HR) and cervical lesions prevalence, describing the distribution of different genotypes, and the epidemiologic clinical characteristics of women co-infected with HIV and HPV (HR). Identify the cytological characteristics in HIV-positive women that frequent the Condesa Specialised Clinic in Mexico City. The cohort was made up of 401 HIV-positive women. The women had a gynaecological exploration, cervical cytology, colposcopy and HPV typfication, and biopsy if necessary. The data was obtained through questionnaires with socio-demographic, behavioural and clinical variables.

**Results:**

HPV (HR) infection prevalence was 34.41%. ASCUS, LSIL and HSIL prevalence was 11.85, 29.63 and 17.78% respectively. With negative genotyping ASCUS was 4.94%, LSIL was 25.1% and HSIL was 4.56%. The more frequent genotypes were HPV NO-16 NO-18 (63.04%), HPV16 (16.67%) and HPV18 (13.04%). Factors associated to HPV (HR) were: age, last PAP abnormal. Factors associated to cytological alterations were: first sexual relation <18 years, last PAP abnormal, CD4 count <200 cells/ml and VL >10,000 copies/ml.

**Conclusions:**

Known factors associated with HIV infection such as low CD4 count and a high viral load are predictors of cytological alterations in this group of patients. There is a high prevalence of HPV (HR) infections and genotypes with a high oncological risk in the study group. Behavioural factors such as age of onset for sexual activity may play a role in these conditions.

## References

[1] Palefsky JM, Minkoff H, Kalish LA, Levine A, Sacks HS, Garcia P (1999). Cervicovaginal human papillomavirus infection in human immunodeficiency virus-1 (HIV)-positive and high-risk HIV-negative women. J Natl Cancer Inst.

[2] Walboomers JM, Jacobs MV, Manos MM, Bosch FX, Kummer JA, Sha KV (1999). Human papillomavirus is a necessary cause of invasive cervical cancer worldwide. J Pathol.

[3] Tirado-Gómez LL, Mohar-Betancourt A, López-Cervantes M, García-Carrancá A, Franco-Marina F, Borges G (2005). Factores de riesgo de cáncer cervicouterino invasor en mujeres mexicanas. Salud Pública Méx.

# Sub article: P013 Clinical presentation and outcomes of HIV-positive patients with diagnosis of tuberculosis at Guillermo Almenara Hospital in Lima, Peru

**DOI:** 10.7448/IAS.18.3.20144

**Published:** 2015-04-16

**Authors:** Oscar Malpartida, Giancarlo Perez, Romulo Rodriguez, Julio Maquera, Ricardo Illescas, Lourdes Rodríguez, Jose Hidalgo

**Affiliations:** Internal Medicine-Infectology, Hospital Guillermo Almenara Irigoyen, Lima, Peru

## Abstract

**Introduction:**

Tuberculosis (TB) continues to be a major cause of morbidity and mortality in HIV patients globally, and this is particularly true in Peru. Our objectives are to describe the frequency, clinical characteristics and outcomes of tuberculosis in HIV/TB co-infected patients at a tertiary care hospital in Lima, and compare these findings among patients receiving highly active antiretroviral therapy (HAART) chronically (Group 1), recently started (Group 2) and not receiving HAART (Group 3).

**Materials and methods:**

Retrospective review of medical records of HIV patients who developed TB during 2014 at Guillermo Almenara Hospital in Lima, Peru. Group 1 included patients receiving HAART for >180 days. Short-term mortality was defined as death during hospitalization or within 30 days post-discharge.

**Results:**

There were 23 cases of TB during the period of study. Affected patients were 87% male with a mean age of 37.9 years and a mean CD4 count of 62. Thirteen cases were pulmonary (56.5%). Extra-pulmonary presentations (10/23, 43.5%) included one CNS involvement (4%), one gastro-intestinal disease (4%) and three multi-organ involvements (13.04%). Twenty-one diagnoses were confirmed microbiologically, 34.7% was MDR and one case was XDR. There were seven (30.4%) patients in Group 1, four (17.4%) in Group 2 – one associated to immune reconstitution – and 11 (47.8%) in Group 3 – associated with recent HIV/AIDS diagnoses. Short-term mortality was significant only in Group 3 (27.3%). Time to TB diagnosis averaged 18.8 days, and diagnoses delays were related to complicated or atypical presentation or lack of access to microbiological confirmation.

**Conclusions:**

TB significantly affected HIV-positive patients. Short-term mortality was high in patients not receiving HAART. Although a relatively low proportion of cases had microbiologically confirmed diagnoses, drug resistance was documented for a high number of cases and higher than other Latin-American countries.

# Sub article: P014 Improving early HIV diagnosis by increasing HIV testing: experience in León, Guanajuato, Mexico

**DOI:** 10.7448/IAS.18.3.20145

**Published:** 2015-04-16

**Authors:** Juan Luis Mosqueda, Ericka Magdalena León Guerrero, José Álvarez, Santiago Ignacio Godinez Hernández, Emilio Francisco Jimenez Guevara

**Affiliations:** 1 Infectology, CAPASITS León, Secretaría de Salud de Guanajuato, León, Guanajuato, Mexico; 2 Departamento de Medicina y Nutrición, Universidad de Guanajuato, León, Guanajuato, Mexico; 3 Investigación, Hospital Regional de Alta Especialidad del Bajío., León, Guanajuato, Mexico

## Abstract

**Introduction:**

Early diagnosis in HIV represents a public health issue. In spite of disease-awareness strategies and follow up in HIV patients, there is a large proportion of individuals that seek medical care in an advanced stage [1–3]. The aim of the study was to determine the temporal trends observed of early diagnosis and the correlation with the number of HIV tests in the city of León, Guanajuato, Mexico.

**Materials and methods:**

All newly diagnosed from 2005 to 2014, ARV naïve, non-insured HIV patients in León, Mexico were included. Baseline CD4+ counts were categorized into four groups: <200 CD4/µl, 201–350 CD4/µl, 351–500 CD4/µl and >500 CD4/µl (early diagnosis). To evaluate the proportion of patients in each category for the evaluated period and the number of HIV tests done for the period of 2008 to 2014 the *X* ^2^ test for trends was performed. The Pearson test was used to make a correlation between early diagnosis and the number of HIV tests.

**Results:**

Of 699 patients, 593 (85%) male were diagnosed during the study period. From 2005 to 2014, the proportion of late diagnosis decreased from a 64 to 31% (*X* ^2^=19.85, *p<*0.0001), the proportion of early diagnosis increased from 5 to a 44% (*X* ^2^=21.9, *p<*0.0001) in the same time period. There was an increase in the number of rapid HIV testing performed per year with a significant trend line (*R* ^2^=0.7685). In the linear regression analysis there was a correlation within the number of the rapid HIV tests effectuated and the percentage of early diagnosis with a correlation coefficient of *R* ^2^=0.8258.

**Conclusions:**

A significant increase in the proportion of early diagnosed HIV patients was observed during the study period in the city of León. A strong correlation was found with the increment of the HIV testing during the same years suggesting widespread testing may have an effect on the clinical stage at presentation.

## References

[1] Crabtree-Ramirez B, Caro-Vega Y, Belaunzaran-Zamudio F, Sierra-Madero J (2012). High Prevalence of late diagnosis of HIV in Mexico during the HAART Era. Salud Püblica Mex.

[2] Carrizosa CM, Blumberg EJ, Hovell MF, Martinez-Donate AP, Garcia-Gonzalez G, Lozada R (2010). Determinants and prevalence of late HIV testing in Tijuana, Mexico. AIDS Patient Care STDS.

[3] Shrosbree J, Campbell LJ, Ibrahim F, Hopkins P, Vizcaychipi M, Strachan S (2013). Late HIV diagnosis is a major risk factor for intensive care unit admission in HIV-positive patients: a single centre observational cohort study. BMC Infect Dis.

# Sub article: P015 HIV-1 subtype distribution and circulating recombinant forms in Colombia 2011–2014

**DOI:** 10.7448/IAS.18.3.20146

**Published:** 2015-04-16

**Authors:** Andres Gonzalez, Daniel Escobar, Jenny Andrea Carmona, Juan Carlos Gonzalez

**Affiliations:** 1 Molecular Biology Laboratory, Dinamica IPS, Medellin, Colombia; 2 School of Medicine, Univesidad Pontificia Bolivariana, Medellin, Colombia; 3 Research Coordination, Dinamica IPS, Medellin, Colombia; 4 Clinica Cardio VID, Scientific Director, Medellin, Colombia

## Abstract

**Introduction:**

Determining the genotype of a virus is important for the development of diagnostics, prophylaxis and effective antiviral treatment. HIV-1 has high degree of diversity and variability: four groups, nine subtypes and seventy-two circulating recombinant forms (CRFs) [1].

**Materials and methods:**

A descriptive, retrospective study, which included sequences of genes protease (PR) and reverse transcriptase (RT) of HIV-1 was performed. Testing was conducted at the Laboratory of Molecular Biology Dinamica IPS Colombia (June 2011 to October 2014). For genotype sequences RT-PR of HIV-1, the TRUGENE HIV-1 Genotyping Assay (Siemens) using the DNA GeneObjects Sequencing System 4.1 software was used [2]. For analysis of HIV-1 subtypes and their CRFs the software of the National Center for Biotechnology Information (NCBI) was employed, using the database Genotyping Reference Dataset (Human Immunodeficiency Virus (HIV-1)) “pure” & CRFs 2005 [3]. The subtypes and recombinant forms were confirmed using the REGA HIV-1 Subtyping Tool software [4,5].

**Results:**

We genotyped and analyzed a total of 301 samples from HIV-1 positive Colombian patients, with failure to antiretroviral therapy. Geographical distribution of the samples: 23 (7.6%) from Bogotá, 123 (40.9%) from Medellin, 135 (44.9%) from Cali and 20 (6.6%) from Barranquilla. In Colombia subtype B was found in 245 (81.4%) of the cases. The recombinant subtype D/B was identified in two cases (0.66%) and six different CRFs from 54 patients were distributed as: 1 (0.3%) case carrying CRF02_AG, 11 (3.65%) cases with CRF03_AB, 2 (0.6%) cases with CRF05_DF, 25 (8.3%) cases with CRF07_BC,7 (2.33%) with CRF08_BC, in 8 (2.66%) cases CRF12_BF was found.

**Conclusions:**

In Colombia subtype B predominates in more than 80%, similar to other South American countries [6]. Argentina, Uruguay and Paraguay report one high prevalence of subtype F. The CRFs most prevalent in Colombia were the CFR07_BC and CFR08_BC with 10.6% of infections. The CFR12_BF is reported in Argentina and Uruguay, this was found in 2.6% of cases in this study. The recombinant form CRF02_AG was the only CRF without recombination of subtype B and has been reported in two cases in Ecuador in 2005; it is commonly found in Africa. The results of this study are useful to understand the diversity of HIV-1 in Colombia and to implement strategies to improve the tools used in the diagnosis and control of this disease.

## References

[1] HIV sequence database HIV circulating recombinant forms (CRFs). http://www.hiv.lanl.gov/content/sequence/HIV/CRFs/CRFs.html.

[2] GenObjects(TM) 4,1 (2008). OpenGene DNA sequencing systems.

[3] National Center for Biotechnology Information (NCBI) Tools, genotyping. http://www.ncbi.nlm.nih.gov/projects/genotyping/formpage.cgi.

[4] De Oliveira T, Deforche K, Cassol S, Salminem M, Paraskevis D, Seebregts C (2005). REGA HIV-1 subtyping tool. An automated genotyping system for analysis of HIV-1 and other microbial sequences. Bioinformatics.

[5] Alcantara LC, Cassol S, Libin P, Deforche K, Pybus OG, Van Ranst M (2009). A Standardized framework for accurate, high-throughput genotyping of recombinant and non-recombinant viral sequences. Nucleic Acids Res.

[6] Laguna-Torres VA, Olson J, Sánchez JL, Montano S, Chauca G, Carrión G (2005). Distribución de los subtipos del VIH-1 en nueve países de América del sur, 1995–2002. Rev Peru Med Exp Salud Publica.

# Sub article: P016 Utility of enzyme-linked immunosorbent assay for detects *Histoplasma capsulatum* antigenuria-like screening in patients with HIV/AIDS

**DOI:** 10.7448/IAS.18.3.20147

**Published:** 2015-04-16

**Authors:** Claudia Frola, Omar Sued, Florencia Straminsky, Liliana Guelfand, Héctor Pérez, Pedro Cahn

**Affiliations:** 1 Infectious Disease Service, Microbiology Laboratory, Hospital Juan A Fernández, Buenos Aires, Argentina; 2 Fundación Huésped, Buenos Aires, Argentina; 3 Microbiology Laboratory, Hospital Juan A Fernández, Buenos Aires, Argentina; 4 Infectious Disease Service, Hospital Juan A Fernández, Buenos Aires, Argentina

## Abstract

**Introduction:**

Histoplasmosis is the second most frequently diagnosed systemic fungal infection in Argentina. Among people living with HIV (PLWH) and low CD4 counts (i.e. <100 cells/mm^3^), it presents as a disseminated disease. Microscopic examination and culture remain today as the reference methods for diagnosis. However, their sensitivity is limited and culture might take some weeks. In this context the detection of urinary antigen (UAg) for *Histoplasma capsulatum* might represent an important advance for the diagnosis. The goal of our study was to explore the aggregated value of the routine detection of UAg among HIV patients with advanced infection, without ART or failing the current regimen.

**Materials and methods:**

Between April and December 2014, PLWH with ≤100 CD4/µl on follow-up in a major public HIV centre in Buenos Aires, Argentina, underwent routine screening of UAg through of an antigen capture enzyme-linked immunosorbent assay (ELISA, IMMY). A cut-off of 0.5 units was considered positive for histoplasmosis. In addition, lysis-centrifugation fungal blood culture and antibodies detection using immunodiffusion were performed. For patients with skin lesions direct examination and culture were also performed.

**Results:**

In total, 114 patients were included: 63.1% were male, the majority was currently off ART (78.9%) and the median CD4 count was 44 cells/µl (IQR 18–81). With the standard algorithm, seven patients were diagnosed as having histoplasmosis (three had positive blood culture plus histopathological diagnosis, two had only histopathological diagnosis while the other two had only blood culture diagnosis). Only one sample had positive serology. Four additional patients were identified with UAg positive. Two of them were symptomatic and improved with itraconazole, the remaining two were lost to follow up. When we included UAg positive samples as cases the prevalence of histoplasmosis increased from 6.1 to 9.6%. Sensitivity, specificity, VPP and VPN were 71, 96, 63 and 98%, respectively.

**Conclusions:**

UAg increased the diagnosis rate of histoplasmosis and has higher sensitivity than other serological tests based on antibodies. These preliminary findings suggest that screening for histoplasmosis infection among PLWH and advanced disease in our setting might be an effective strategy to improve clinical care of PLWH.

# Sub article: P017 Low virologic failure on TDF/FTC/RPV in HIV-infected naïve and virologically suppressed patients with strict clinical selection and/or DNA genotyping

**DOI:** 10.7448/IAS.18.3.20148

**Published:** 2015-04-16

**Authors:** Camille Bernaud, Audrey Rodallec, Marine Morrier, Clotilde Allavena, Philippe Perre, Solene Pineau, Thomas Jovelin, Nolwenn Hall, Samuel Pineau, Elisabeth Andre-Garnier, Francois Raffi

**Affiliations:** 1 Infectious Diseases, University of Nantes, Nantes, France; 2 Virology, University of Nantes, Nantes, France; 3 Infectious Diseases, Testing Centers CDAG HIV/AIDS, La Roche sur Yon, France

## Abstract

**Introduction:**

TDF/FTC/RPV has advantages among STR including greater convenience, tolerability and lower drug-drug CYP3A interactions. Main drawbacks are need for food intake, concomitant PPI contra-indication and compromised antiviral activity when various NNRTI mutations are present. We evaluated TDF/FTC/RPV in a real life setting with focus on clinical and virological vigilance.

**Methods:**

OCEAN II is a prospective, two-centre observational study. From September 2012 to December 2013, all antiretroviral naïve patients with HIV RNA <100,000 copies/ml or wish to switch for simplification were considered for treatment with TDF/FTC/RPV. A systematic review of potential obstacles to TDF/FTC/RPV administration was undertaken, including food requirement, PPI co-administration, physician's issues regarding adherence, and in case of undetectable plasma HIV RNA, DNA genotyping to detect archived RPV and/or NRTI-associated resistance mutations.

**Results:**

TDF/FTC/RPV was discussed in 498 patients: TDF/FTC/RPV was not offered to 194 patients (39%), mainly for NNRTI or NRTI resistance on genotypic testing (historical RNA or current proviral DNA) and/or history of virologic failure on dual NRTI therapy or NNRTI-containing regimen (*n*=55), issues on adherence (*n*=35), patient refusal to change their current regimen (*n*=31), difficulties to take treatment with meals (*n*=18) or concomitant PPI therapy (*n*=14). The 304 patients treated with TDF/FTC/RPV (285 switch; 19 naïve with mean HIV RNA of 31,000 c/ml) had a median CD4+ cell count of 632/µl. After median follow-up of 24 months, virological failure occurred in three patients (1%), all switch, with emergence of resistance mutations in 1/3, while two patients experienced rebound after transient treatment interruption, and further control after treatment resumption. Median decrease in eGFR (MDRD) at M12 was –8.23 ml/min (range –35 to +29.5). Discontinuation of TDF/FTC/RPV occurred in 67 patients: gastro-intestinal disorders (*n*=13), drug interaction (*n*=11), creatinine increase (*n*=9), pregnancy (*n*=8), other adverse events (*n*=13). No adverse events were grade 3 or 4.

**Conclusions:**

TDF/FTC/RPV is suitable for most patients. Strict clinical and virologic screening was associated with a low risk of virologic failure. In this real-life experience, 22% of patients stopped therapy during two years of follow-up, most frequently for minor events.

# Sub article: P018 An overview of a cohort of elderly patients in Mexico

**DOI:** 10.7448/IAS.18.3.20149

**Published:** 2015-04-16

**Authors:** Luz González-Hernández, Mara Llamas-Covarrubias, Raul Soria-Rodríguez, Emmanuel Mercado-Nuñez, José Martínez-Olmedo, Jaime Andrade-Villanueva

**Affiliations:** HIV Unit, Hospital Civil de Guadalajara, Guadalajara, Mexico

## Abstract

**Introduction:**

The introduction of highly active antiretroviral therapy (HAART) has significantly improved life expectancy on HIV-infected subjects and the population is becoming older. The CDC considers elderly HIV-patients at the age ≥50 years due to the impact of HIV-infection *per se* and the use of antiretrovirals [1]. Information regarding the elderly-infected population is still limited. The aim of this study was to describe the profile in a cohort of patients ≥50 years followed-up in a HIV-care at Guadalajara, Mexico.

**Materials and methods:**

Patients were retrospectively analyzed at the HIV-unit in Hospital Civil de Guadalajara. Patients ≥50 years currently being followed-up at the site were included. Data were obtained from an electronic database. Significance level was established ≤5%.

**Results:**

A total of 393 patients were included which represent the 17% of the total of patients currently followed-up at the site. Eighty-two percent were male, and age ranged from 50 to 90. Women have a higher Body Mass Index (BMI) than men and are mostly classified as pre-obesity. The 37% of patients were diagnosed at an age ≥50 years, and age at diagnosis was higher in women (mean 47 vs 49, *p*=0.025), but, men were more likely to present with advanced-stage (70% C stage); moreover, the 70% had a CD4-count nadir <200 cells/µl without gender differences. Overall, 88% of patients have virologic control, and although no statistically significant the percentage was higher in men, however, 8% of them remained with a CD4-count ≤200 cells/µl (mean of 8.9 years on-HAART) compared to 5% of women (mean of 7.9 years on-HAART); we found two elite-controller women (*p*=0.032). Smoking index was higher in men (median 6 vs 1.4, *p*=0.004), and there was a higher prevalence of arterial hypertension and depression (31% vs 17% and 32% vs 13%, respectively), with a significant higher VACS index among women (median 28 vs 18, *p*=0.003).

**Conclusions:**

Contrary to the reports on young women, it seems that old women reached a good virologic response and immune reconstitution percentages as well as old men. The higher BMI and the association of the VACS Index to markers of chronic inflammation and hypercoagulability [2] may pose special risks for cardiovascular diseases on women and tobacco use and more advanced stages for men. Our population is young, but we need to do early intervention in modifiable cardiovascular risk factor.

## References

[1] López-Otín C, Blasco MA, Partridge L, Serrano M, Kroemer G (2013). The hallmarks of aging. Cell.

[2] Justice AC, Modur SP, Tate JP, Althoff KN, Jacobson LP, Gebo KA (2013). Predictive accuracy of the Veterans Aging Cohort Study index for mortality with HIV infection: a North American cross cohort analysis. J Acquir Immune Defic Syndr.

# Sub article: P019 Prevalence of cardiovascular risk factors in persons living with HIV at high activity antiretroviral therapy in Mexico City

**DOI:** 10.7448/IAS.18.3.20150

**Published:** 2015-04-16

**Authors:** Marisol Valenzuela Lara, Carlos Magis Rodríguez

**Affiliations:** Integral Care Direction, Censida, Mexico City, Mexico

## Abstract

**Introduction:**

The success of high activity antiretroviral therapy (HAART) is associated with the “ageing” of the HIV epidemic [1]. In Mexico 15.7% of people living with HIV (PLH) receiving HAART at the Mexican Health Department (MHD) are aged 50 years or older [2]. Health-care delivery systems must be ready to provide quality integral treatment to PLH, making it necessary to study the different cardiovascular risk factors among this particular population [3,4].

**Material and methods:**

A cross-sectional study was conducted using a database built from chart review and laboratory's database of Condesa Clinic with data from 2009 to 2012. The clinic is in Mexico City and is the largest clinic in Mexico. The objective was to describe the prevalence of cardiovascular risk factors in PLH in HAART age 20 years or older. Age, gender, time since diagnosis, high blood pressure (BHP, SP≥140 mmHg, DP≥90 mmHg), diabetes mellitus (DM≥126 mg/d), hypercholesterolemia (≥200 mg/dl), hypertriglyceridemia (≥150 mg/dl), smoking and obesity (BMI≥30 kg/m^2^) and Framingham risk score were studied. Gender was categorized in men, women and transwomen. The database was validated and analyzed using Stata11.

**Results:**

A total of 904 men, 64 women and 28 transwomen were analyzed. Mean age was 36.5 (95% CIs 35.9–37.1), glucose level 96.8 mg/dl (95% CIs 95.2–98.5), cholesterol 171.5 mg/dl (95% CIs 168.3–174.7), HDL 37.8 mg/dl (95% CIs 37.0–38.6), triglycerides 239.3 mg/dl (95% CIs 227.8–250.9). Framingham risk score was 4.9% (95% CIs 4.6–5.1). BMI significantly increased 1.8 kg/m^2^ (95% CIs 1.7–2.1). Prevalence were hypercholesterolemia 25.8% (95% CIs 23.1–28.5), hypertriglyceridemia 59.1% (95% CIs 56.1–62.2), DM 3.2% (95% CIs 2.1–4.3), HBP 11.1% (95% CIs 9.2–13.1), smoking 42.1% (95% CIs 39.0–45.1), overweight/obesity 44.1% (95% CIs 41.0–47.2). The 23.6% (95% CIs 21.0–26.2) of PLV had moderate risk (5<10%), 7.0% (95% CIs 5.6–9.0) had high risk (10<15%) and 3.3% (95% CIs 2.2–4.4) had very high risk (≥15%). All the prevalence increased in direct proportion with age.

**Conclusions:**

Prevalence of hypertriglyceridemia and smoking were significantly higher than the nationals reported in the Mexican National Health and Nutrition Survey (31.5%, 19.9%). Hypercholesterolemia, DM, HBP and obesity were lower than national prevalence. Ageing is a key factor in the risk to cardiovascular events, but timely intervention at early ages to modify some risk factors could be beneficial in the long term.

**Table:** Abstract P019

	Whole population *n*=996	20–29 *n*=244	30–39 *n*=417	40–49 *n*=238	≥0 *n*=97
Hypercholesterolemia*	25.8 (23.1–28.5)	16.8 (12.1–21.5)	21.3 (17.4–25.3)	33.6 (27.6–39.7)	48.5 (38.3–58.6)
Hypertriglyceridemia*	59.1 (56.1–62.2)	39.8 (33.6–45.9)	59.5 (54.7–64.2)	70.2 (64.3–76.0)	79.4 (71.2–87.6)
Diabetes Mellitus*	3.2 (2.1–4.3)	0.0	1.7 (0.4–2.9)	7.1 (3.8–10.4)	8.3 (2.7–13.8)
High blood pressure*	11.1 (9.2–13.1)	6.6 (3.4–9.7)	7.7 (5.1–10.2)	16.8 (12.0–21.6)	23.7 (15.1–32.3)
Smoking*	42.1 (39.0–45.1)	47.1 (40.8–53.4)	45.3 (40.5–50.1)	36.1 (30.0–42.3)	29.9 (20.6–39.2)
Nutritional status					
Overweight (25<30 kg/m^2^)*	36.4 (33.4–39.3)	20.1 (15.1–25.1)	37.9 (33.2–42.6)	48.3 (41.9–54.7)	41.2 (31.3–51.2)
Obesity (≥30 kg/m^2^)*	7.7 (6.1–9.4)	5.7 (2.8–8.7)	6.0 (3.7–8.3)	10.9 (6.9–14.9)	12.4 (5.7–19.0)
Framingham					
Moderate (5<10%)*	23.6 (21.0–26.2)	5.7 (2.8–8.7)	19.4 (15.6–23.2)	44.1 (37.8–50.5)	36.1 (26.4–45.8)
High (10<15%)*	7.0 (5.6–9.0)	0.0	2.9 (1.3–4.5)	12.6 (8.4–16.9)	28.9 (19.7–38.0)
Very high (≥15%)*	3.3 (2.2–4.4)	0.0	0.2 (0.0–1.3)	2.9 (0.8–5.1)	25.8 (16.9–34.6)

Data are presented as percentages.

* *p<*0.05.

## References

[1] UNAIDS (2013). HIV and aging.

[2] Censida (2014). System of management, logistic and monitoring of antiretroviral [database].

[3] Deeks SG, Lewin SR, Havlir DV (2013). The end of AIDS: HIV infection as a chronic disease. Lancet.

[4] Kelesidis T, Currier JS (2014). Dyslipidemia and cardiovascular risk in human immunodeficiency virus infection. Endocrinol Metab Clin North Am.

# Sub article: P020 Death causes in HIV patients between 1985 and 2013 in Infectious Diseases Service of Centro Hospitalar do Porto, Portugal

**DOI:** 10.7448/IAS.18.3.20151

**Published:** 2015-04-16

**Authors:** Celina Gonçalves, Miguel Abreu, Ana Rita Silva, Josefina Mendez, Rui Sarmento e Castro

**Affiliations:** 1 Serviço de Infecciologia, Centro Hospitalar do Porto, UJU, Porto, Portugal; 2 Serviço de Infecciologia, Hospital de Loures, Porto, Portugal

## Abstract

**Introduction:**

After the introduction of effective antiretroviral therapy (highly active antiretroviral therapy (HAART)), the average life expectancy of patients with HIV infection have approached the general population.

**Purpose:**

Analyze the causes of death in HIV patients according to the treatment regimen used.

**Methods and materials:**

Retrospective analysis of medical records of HIV patients who died in our service since 1985. For analysis, patients were divided into three periods, depending on the type of treatment carried out – from 1985 to 1996 (pre-HAART), 1997 to 1999 (partial use of HAART) and 2000 to 2013 (HAART).

**Results:**

Between 1985 and 2013 were observed in our service 4962 HIV-infected patients, of which 1056 died (21%). Of the deaths, 157 (15%) occurred before 1997, 235 (22%) in the period 1997–1999 and 664 (63%) between 2000 and 2013. The mortality rate was, respectively, 19, 12 and 13%. Of the deaths, 86% were male in the pre-HAART period, maintaining a similar proportion in the interim and HAART period (83 and 86%). Considering the average age at the date of death, this was 35 years in the pre-HAART era, 33 in partial use of HAART and 40 years in the HAART period. The average length of knowledge of HIV infection was 20 months, 25 months and 64 months, respectively. The mean nadir CD4 lymphocytes was similar in the three periods: 128, 112 and 96 cells/mm^3^; since the average value of CD4 to the date of death increased from 98 in the pre-HAART period, to 101 in the partial use of HAART and 130 cells/mm^3^ in the HAART era. Only 7% of patients presented suppressed on the death of the height in the pre-HAART era, a percentage that has remained by 13% in the HAART era.

It was possible to identify the causes of death in 889 of the cases (167 in the cause remained undetermined). The characterization is summarized in the table below:

**Conclusions:**

There was a significant fall in the mortality rate of the first and the last years of HIV infection as a result of more effective therapeutic use. The introduction of HAART has led to a change in the cause of death, with relative increase in mortality related with chronic hepatic disease, not opportunistic infections and tumours.

**Table:** 

Death cause	Pre-HAART (%)	Partial use of HAART (%)	HAART (%)
AIDS defining illness	63.9	71.1	51
Non-opportunistic infections	21.1	17.1	28.3
Chronic hepatic disease decompensation	4.5	5.9	12.6
AIDS non-defining tumour	0	0.7	3.5
Others	10.5	5.3	4.6

# Sub article: P021 Impulsivity and depressive symptoms in people with HIV diagnosed with a common mental disorder from an HIV clinic in Mexico City

**DOI:** 10.7448/IAS.18.3.20152

**Published:** 2015-04-16

**Authors:** Hamid Vega-Ramirez, Victor Rodriguez, Jeremy Cruz, Harumi Hirata, Daniela Ferreyra, Carolina Rocabert

**Affiliations:** 1 Mental Health Program, Condesa Specialized Clinic, Mexico City, Mexico

## Abstract

**Introduction:**

Mental disorders are more prevalent in people with HIV [1]. Having a mental disorder interferes with highly active antiretroviral therapy (HAART) adherence. Impulsivity and depressive symptoms have been reported in association with risky sexual behaviour and poor adherence to HAART in people with HIV [2,3]. The main objective of this study was to compare the level of impulsivity and depressive symptoms in a sample of HIV patients diagnosed with a common mental disorder.

**Materials and methods:**

The sample was patients recently diagnosed with HIV at the Condesa Specialized Clinic from 2013 to 2014. A psychiatrist or clinical psychologist, based on the clinical interview and ICD-10 criteria, made the diagnosis of the mental disorder for each patient. Impulsivity and depressive symptoms were measured with adapted self-administered short versions of the State Impulsiveness Scale (SIS) and the Beck Depression Inventory (BDI). We performed ANOVA analysis to compare age, education, viral load, CD4 count, SIS and BDI score between groups.

**Results:**

We obtained a sample of 1350 patients, of whom 89.1% were male, 9.0% were female and 1.9% were transgender women. The mean age was 31.9 (±9.3) years, and the mean of education was 11.9 (±3.6) years. The mean viral load was 268,017 (±934,431) copies/ml, and the count of CD4 was 333 (±255) cells/µl. A total of 40.8% of the sample had no mental disorder, 31.8% adjustment disorder, 13.0% major depressive episode, 4.8% general anxiety disorder, 5.4% ethanol misuse, 2.6% cocaine misuse and 1.6% cannabis misuse. The mean difference of age (*F=*2.28, gl=6, *p*<0.03) and education (*F=*3.27, gl=6, *p*<0.003) were statistically different between groups. The level of impulsivity (*F=*36.6, gl=6, *p*<0.001), the IBD score (*F=*63.4, gl=6, *p*<0.001), viral load (*F=*3.0, gl=6, *p*<0.005) and CD4 count (*F=*2.9, gl=6, *p*<0.006) were also statistically different. The highest means of SIS (12.7 (±8.9)) and IBD (12.3 (±6.9)) scores were found in the group with a MDE, and lowest scores in the group were found with no mental disorder (5.1 (±5.3); 3.4 (±4.5)).

**Conclusions:**

This study suggests that HIV patients with high levels of impulsivity and depressive symptoms are aligned with their clinical mental diagnosis. This could be helpful in clinical practice to identify those HIV patients with impulsivity and depressive symptoms that are at greater risk to have unprotected sex or poor HAART adherence.

## References

[1] Bing E, Burnam MA, Longshore D, Fleishman JA, Sherbourne CD, London AS (2001). Psychiatric disorders and drug use among human immunodeficiency virus–infected adults in the United States. Arch Gen Psychiatry.

[2] Cook JA, Grey D, Burke J, Cohen MH, Gurtman AC, Richardson JL (2004). Depressive symptoms and AIDS-related mortality among a multisite cohort of HIV-positive women. Am J Public Health.

[3] Gonzalez JS, Batchelder AW, Psaros C, Safren SA (2011). Depression and HIV/AIDS treatment nonadherence: a review and meta-analysis. J Acquir Immune Defic Syndr.

# Sub article: P022 Reliability, structure and factorial congruence of the State Impulsivity Scale in men of an HIV specialized clinic in Mexico City

**DOI:** 10.7448/IAS.18.3.20153

**Published:** 2015-04-16

**Authors:** Victor Rodriguez, Hamid Vega-Ramirez, Jeremy Cruz, Daniela Ferreyra, Gabriela Hernandez, Carolina Rocabert

**Affiliations:** Mental Health Program, Condesa Specialized Clinic, Mexico City, Mexico

## Abstract

**Introduction:**

State Impulsivity (SI) is a tendency to respond quickly in an unanticipated and unplanned fashion to specific environmental situations. In the HIV population, it is important to detect pathological SI to predict treatment adherence and risk of reinfections. Today there are questionnaires like Barratt or Plutchik scales, but these consider impulsivity as psychopathic behaviours rather than a personality feature [1–3]. These SI instruments have not been validated in Mexican population. Therefore, we obtained the internal consistency, construct validity and factorial congruence of the State Impulsivity Scale (SIS) in Mexican men with HIV [4].

**Materials and methods:**

We conducted a cross-sectional study in 488 HIV-positive men (35.4±12.4 years old, 12.7±3.6 years of education and 5.6±5.0 years of diagnosis). After signing informed consent, participants completed the SIS and demographic data. The analysis of SIS psychometric properties was made using standard procedures: reliability with Cronbach's coefficient and the inter-item correlations to define the rotation method for the factor analysis. The item's lowest limit load to be included was 0.40. The factorial congruence between the two factorial loads (original version and this study) was tested with Wrigley and Nauhaus coefficient. The statistical analysis was performed in SPSS *v*. 20, considering *p<*0.01 significant [5].

**Results:**

Internal consistency analysis obtained a mean Cronbach's alpha of 0.92. Using factor analysis with oblique rotation (mean inter-item correlations *r=*0.71, KMO=0.93), we found two factors: attentional-automatism and gratification (17 of 20 items). These factors explained 42.1 and 6.9% of the variance, respectively. The subsequent analysis of internal consistency indicated a total coefficient of 0.84 to 0.91 for each factor. The factorial congruence coefficients between the two versions of SIS (original and adapted) ranged from 0.598 to 0.971 (Table 1).

**Conclusions:**

We demonstrate that the adapted version of SIS for Mexican population is reliable, valid and conceptually equivalent for patients with HIV. The coefficients obtained were higher than those obtained in the original Spanish version. The SIS is useful for measuring impulsivity factors related to the therapeutic adherence and reinfection risk (e.g. drug use and unsafe sexual practices) reported in other studies.

**Abstract P022–Table 1:** Psychometric properties of SIS

Factor (items)	Mean score (0–3 rank)	Cronbach's α	Explained variance (%)	Item-total correlations	Factorial congruence coefficients
Automatism attentional (12)	1.44	0.91	42.1	0.52–0.75	0.708–0.971
Gratification (5)	0.65	0.81	6.9	0.40–0.69	0.598–0831
SIS total		0.92	49.0		

## References

[1] Iribarren MM, Jiménez M, García JM, Rubio G (2011). Validation and psychometric properties of the State Impulsivity Scale (SIS). Actas Esp Psiquiatr.

[2] Páez F, Jiménez A, López A, Raull J, Ortega H, Nicolini H (1996). Estudio de validez de la traducción al castellano de la Escala de Impulsividad de Plutchik. Salud Ment.

[3] López PL, Cetkovich M, Lischinsky A, Alvarez D, Torrente F (2012). Psychometric properties of the Barratt Impulsiveness Scale in a sample of Buenos Aires. Vertex.

[4] Monterosso J, Ainslie G (1999). Beyond discounting: possible experimental models of impulse control. Psychopharmacology.

[5] Nunnally JC, Bernstein YJ (1994). Psychometric theory.

# Sub article: P023 Dyslipidemia and use of lipid-lowering agents in Mexican HIV-positive patients using boosted atazanavir and boosted lopinavir

**DOI:** 10.7448/IAS.18.3.20154

**Published:** 2015-04-16

**Authors:** Gisela Munoz, Ismael Avila, Luis Soto-Ramírez

**Affiliations:** Infectologia, Instituto Nacional De Ciencia Medicas Y Nutricion Salvador Zubiran, Mexico City, Mexico

## Abstract

**Introduction:**

Lipid abnormalities in patients on HAART have been associated with use of boosted protease inhibitor (bPI), but some of them, such as atazanavir, are considered more lipid-friendly. Our aim was to investigate the effects of boosted atazanavir (ATV/r) and lopinavir (LPV/r) on lipids, and the proportion of patients who need lipid-lowering agents (LLA), in Mexican HIV-positive patients.

**Materials and methods:**

We conducted a retrospective cohort study in patients followed at the HIV clinic of the INCMNSZ in Mexico City. Patients receiving LPV/r or ATV/r for at least one year, and having a complete lipid profile, were included. History of antiretroviral therapy (ART), use of LLA and triglycerides (TG), total cholesterol (TC), HDL and LDL were collected at baseline and after one year of the start of PI/r. The primary endpoint was changed in TC, LDL and HDL and TG, and proportion of patients who needed to use LLA. We considered severe dyslipidemia (SD) TC values ≥240 and/or TG≥500 mg/dl.

**Results:**

A total of 101 (14.8% female, median age 44 [21–75]) patients were evaluated: 48 (47.52%) received ATV/r and 53 (52.47%) LPV/r. Sixteen and fourteen patients received ATV/r and LPV/r, respectively, as first line treatment, whereas 32 and 39 had ATV/r and LPV/r as salvage. At baseline, in patients with ATV/r, 16 (33%) were using LLA and 14/48 (29%) had SD, while in those on LPV/r, 14 (26%) were using LLA and 4/53 (7.5%) had SD. Thirty (29%) patients started LLA during treatment, with a median duration of 6.76 mo., most of them fibrates (83%); 16 (53%) belonged to ATV/r, and 14 (46%) to LPV/r (*p=*0.23). At the end of the study, TC levels increased by 16.88% from baseline with LPV/r regardless of the use of LLA, while a slight decrease by 10.75% was seen for ATV/r. However, these values did not surpass normal limits. LDL increased slightly with ATV/r and LPV/r, without reaching abnormal values. No statistical difference was found between the groups for TC and LDL. TG and HDL increased significantly for both drugs between baseline and one year (*p*≤0.001). However, no difference was seen in mean changes in TG (*p=*0.10) and HDL (*p=*0.18) between the two drugs. Regarding SD, the number of cases in the ATV/r group decreased significantly in patients without LLA (*p=*0.05); however, the decrease was not significant (*p=*0.48) in patients receiving LLA. In the group with LPV/r, no significant differences were seen. No significant difference was found between groups.

**Conclusions:**

The main lipid alteration seen in our study was hyper TG, related to the use of ritonavir and not different between the two bPI studied. No significant differences in lipid profiles, use of LLA nor in presence of SD were found between both ATV/r and LPV/r, especially in cases with dyslipidemia before treatment. For cases with lipid abnormalities before treatment, other lipid-friendly drugs should be preferred.

# Sub article: P024 Evaluating the use and reporting of minority variants in HIV genotypic testing using ultra-deep sequencing

**DOI:** 10.7448/IAS.18.3.20155

**Published:** 2015-04-16

**Authors:** Leonardo Chanqueo, Howard Martin, Kim Brugger, Aws Sadik, Hongyi Zhang, Jane Greatorex

**Affiliations:** 1 Department of Medicine, University of Cambridge, Cambridge, UK; 2 Hospital San Juan De Dios, Santiago, Chile; 3 Clinical Translational Research Unit, East Anglian Medical Genetics Service, Cambridge, UK; 4 East Anglian Medical Genetics Service, Cambridge, UK; 5 Public Health England, Addenbrooke's Hospital, Cambridge, UK

## Abstract

**Introduction:**

Ultra-deep sequencing (UDS) allows the detection of HIV-1 drug resistance mutations with prevalence <20%. However, there are conflicting data on the utility and interpretation of reporting low frequency mutations. Using UDS with two different cut-offs compared to standard capillary sequencing, the aim of this study was to evaluate the changes in HIV genotypic test reports and their potential impact on patient care.

**Materials and methods:**

Over a 12 month period, 68 plasma samples from in-patients were collected and extracted on a QiaSymphony and amplified using an in-house polymerase chain reaction (PCR)-based assay. DNA was cleaned up and quantified prior to UDS (Illumina MiSeq^®^). Data was analyzed and a report generated using a bespoke pipeline, reporting drug resistance mutations (DRMs) at cut-offs of 1 to 1.99%, 2 to 19.9% and >20%. Clinical details were obtained with consent from in-patient test request forms. Virus strains included 30 subtype B, 24 subtype C and 14 non B or C subtypes; patients were drug-naïve (*n*=43), treatment failures (*n*=15) and “other” (*n*=10); viral loads ranged between 180 and 3.2×10^6^ gc/ml.

**Results:**

Overall, a significant increase in resistance to antiretroviral therapy (ART) was detected using the more sensitive sequencing technology. A>2% cut-off resulted in a change to the resistance report from susceptible to resistant for 5/68 patients; a>1% cut-off increased this number to 22/68. Using established criteria, transmitted drug resistance was 19% with the 2% cut-off and 41% with the 1% cut-off, compared to 14% with traditional Sanger sequencing (20% cut-off). DRMs were more frequent in patients for whom ART therapy was failing. All classes of ART had a 10 to 13% increase in DRM detected.

**Conclusions:**

The UDS assay performed well across a wide range of subtypes, viral loads and different populations of patients, increasing the detection of minority variants, especially DRMs at a 1 to 1.99% cut-off. This increase in detected DRMs led overall to a change in 22% of HIV genotyping reports. Taking this into account, it is suggested that a 1% cut-off should be routinely utilized. The clinical implications of reporting the variants will be discussed; however, further studies are needed in order to assess what the full impact of reporting these minority variants will be on treatment efficacy.

# Sub article: P026 Week 24 data from a phase 3 clinical trial of E/C/F/TAF in HIV-positive adolescents

**DOI:** 10.7448/IAS.18.3.20157

**Published:** 2015-04-16

**Authors:** Jag Batra, Hilda Kizito, Aditya Gaur, Wasana Prasitsuebsai, Natella Rakhmanina, Eileen Lawson, Yongwu Shao, Sean Bennett, Andrew Cheng, Anita Silva, Erin Quirk

**Affiliations:** 1 Brickstaff Family Center, Miller Children's Hospital, Long Beach, CA, USA; 2 Joint Clinical Research Centre, Kampala, Uganda; 3 Department of Infectious Diseases, St. Jude Children's Research Hospital, Memphis, TN, USA; 4 Thai Red Cross AIDS Research Centre (TRC-ARC), HIVNAT, Bangkok, Thailand; 5 Infectious Disease Department, Children's National Health System, Washington, DC, USA; 6 Clinical Research, Gilead Sciences, Foster City, CA, USA; 7 Public Health & Medical Affairs, Gilead Sciences Farmaceutica do Brasil, Sao Paulo, Brazil

## Abstract

**Introduction:**

EVG/COBI/FTC/tenofovir alafenamide (TAF) (E/C/F/TAF) is an integrase-inhibitor-based single-tablet regimen in clinical development for use in HIV-positive adolescents. Pharmacokinetics, safety and efficacy from a planned interim analysis of the first clinical trial of E/C/F/TAF in adolescents are reported.

**Materials and methods:**

Treatment-naïve 12 to <18 year-olds weighing ≥35 kg with HIV-1 RNA >1000 copies/ml (c/ml), CD4 >100 cells/µl and eGFR >90 ml/min/1.73 m^2^ received E/C/F/TAF once daily in a prospective, two-part, 48-week, single-arm, open-label trial. Steady-state pharmacokinetic (PK) parameters were compared to an adult reference population by ANOVA and related to the range of exposures associated with antiviral activity in adults. Adverse events (AEs), laboratory tests and the proportion of subjects with HIV-1 RNA<50 c/ml were assessed through Week 24. Bone mineral density (BMD) was measured by dual-energy X-ray absorptiometry.

**Results:**

The trial enrolled 48 adolescents with a median age of 15 years, median weight of 52 kg, 58% female, 88% Black, 13% Asian, 67% vertically infected, 35% with HIV-1 RNA >100,000 c/ml, median CD4 count 468 cells/µl and median serum creatinine (sCr) 0.57 mg/dl. TAF, TFV, EVG, COBI and FTC PK profiles of adolescents were consistent in adults. Of 23 subjects followed to Week 24, 21 (91%) had HIV-1 RNA <50 c/ml (Figure 1). No deaths or AE-related discontinuations occurred. The most frequent AEs were nausea (23%), upper respiratory infection (21%) and diarrhoea (17%). One serious AE of visual impairment and intermediate uveitis occurred and resolved without interruption of E/C/F/TAF. The median change in sCr was +0.08 mg/dl at Week 24, consistent with cobicistat's inhibition of renal tubular Cr secretion. No renal failure or proximal renal tubulopathy occurred. From baseline to Week 24, the change in median spine BMD was +2.8% with a change in height-adjusted (HA) Z-score of +0.02 and 2/23 subjects (9%) having a decrease of ≥4%. The change in median total body less head BMD was +0.3% with a change in HA Z-score of +0.09 and no decreases of ≥4%. No fractures occurred.

**Conclusions:**

Therapeutic plasma concentrations of all components of E/C/F/TAF were achieved, consistent with potent antiviral activity of the regimen. Treatment was generally well tolerated through 24 weeks with a favourable renal and bone safety profile. These promising findings support E/C/F/TAF's eventual use in adolescents and its further evaluation in other paediatric populations.

**Abstract P026–Figure 1:** E/C/F/TAF in adolescents: virologic success and CD4 recovery.

# Sub article: P027 Emergence of drug resistance mutations in a cohort of HIV-infected children with mother to child transmission, during follow-up

**DOI:** 10.7448/IAS.18.3.20158

**Published:** 2015-04-16

**Authors:** Cristina de Jesus Herrera-Castillo, Angelica Rodríguez, Othon Rojas-Montes, Rosalia Lira, José Guillermo Vazquez-Rosales

**Affiliations:** 1 Department of Virology, UMAE Hospital of Paediatrics CMN Siglo XXI IMSS, Mexico City, Mexico; 2 Department of Infectology, UMAE Hospital of Paediatrics CMN Siglo XXI IMSS, Mexico City, Mexico

## Abstract

**Introduction:**

In children with HIV vertical transmission infection, successful response to antiretroviral therapy (ART) depends on the previous susceptibility to antiretroviral drugs. In consequence, the virological failure (VF) may emerge due to the presence of drug-resistant viruses and clinical outcome could be deleterious. In low-resource countries, the causes of VF are not routinely assessed by genotyping assays, resulting in a continuous failure to different ART combinations. The aim of the study was to describe the emergence of resistance mutations (RMs) during the follow-up of HIV-1-infected children with VF, compared to patients with successful treatment.

**Material and methods:**

Longitudinal study including plasma samples from 37 vertically infected HIV children collected between 1998 and 2011. Eighteen basal samples were obtained before starting treatment from patients that responded to ART, and 57 samples were obtained from 19 patients with VF in different time points during the follow-up. The samples were stored at −70°C, until the genotypic analysis was performed. A nested RT-PCR was utilized to amplify a fragment of 1084 bp, including protease and reverse transcriptase (RT) sequence. The resistance genotype was determined using a Stanford Genotypic resistance interpretation algorithm.

**Results:**

In basal samples, none of the successfully treated patients had protease inhibitor (PI) drug resistance mutations (DRM) compared to patients with VF (26% had PI DRM). With respect to RT inhibitors (NRTI/NNRTI), 44% of the responders had RT DRM (NRTI (28%), and NNRTI (16%)), and for the VF group, 47% (NRTI (32%) NNRTI (16%)). During the follow-up of the patients with VF, 63% acquired new PI DRM (I54V (58%), V82A (53%), M46I (23%), I84V (21.1%)). Only 16% did not have acquired RT DRM, but 84% developed NRTI DRM (M184V (74%), T215 (42%) D67N (37%), K70R (32%), K219Q (26%)) and 37% developed NNRTI DRM (Y181C, K103N and K101E, 10% each one). Average time to PI DRM emergency was 11 to 34 months, for NNRTI was 2 to 26 months, and for NRTI was 12 to 37 months.

**Conclusions:**

HIV-infected children with transmitted PI DRM had a higher risk to developed VF (OR 8.88). Transmission of RT DRM was not associated to the VF (OR 0.91). During the follow-up, more new PI DRMs were observed in those patients with transmitted PI DRM from the infected mother. Genotyping assay is recommended in naïve paediatric patients to reduce risk of treatment failure.

# Sub article: P028 Effectiveness of an antiretroviral regimen based on genotyping data in HIV-1 highly experimented children to antiretroviral therapy

**DOI:** 10.7448/IAS.18.3.20159

**Published:** 2015-04-16

**Authors:** Gloria Huerta-García, José Guillermo Vazquez-Rosales, Leoncio Peregrino-Bejarano, Eric Flores-Ruiz, Fortino Solorzano-Santos, José Antonio Mata-Marín

**Affiliations:** 1 Department of Pediatric Infectious Disease, UMAE Hospital de Pediatría CMN SXXI, Mexico City, Mexico; 2 UMAE Hospital de Pediatría CMN SXXI, Mexico City, Mexico; 3 Department of Infectious Diseases, Hospital de Infectología. Centro Medico Nacional “La Raza”, Mexico City, Mexico

## Abstract

**Introduction:**

Genotyping tests were developed to help attenuate the impact of viral resistance. These tests can detect mutations associated with phenotypic resistance of HIV to antiretrovirals [1]. Data on the use of ritonavir-boosted darunavir (DRV/r), tipranavir (TPV/r), etravirine (ETV) and/or raltegravir (RAL) for children in resource-limited settings are rare [2].

**Materials and methods:**

We conducted a retrospective cohort study, which included 16 children with virological failure and triple-class drug HIV resistance HIV-1 infection. All patients had protease and retrotranscriptase genotype, and they were evaluated using the Stanford HIV database for resistance mutation interpretation. Switch of antiretroviral (ARV) regimen was based on genotyping data, evaluated by an experts group in antiretroviral drugs resistance. The primary end point was virologic suppression (<50 copies/ml) and immunologic improvement at Week 24. Median and interquartile range (IQR) were used in descriptive analysis.

**Results:**

A total of 16 children were enrolled. The children's median age was 14.5 (IQR 11 to 16.5). Baseline median CD4+ cell count was 382 cells/mm^3^ (IQR 281 to 687) and median plasma HIV-RNA viral load was 15,855 copies/ml (IQR 2952 to 77,089). New drugs such as darunavir (13/16), tipranavir (3/16), raltegravir (13/16) and etravirine (3/16) were included in the new regimen. Median increased in CD4+ cells count to 640 cells/mm^3^ after 24 weeks of regimen based on genotyping data (*p=*0.001). In 11 children (68.8%), plasma HIV-1 RNA viral load was <50 copies/ml, with median HIV-1 RNA viral load of 21 copies/ml (IQR 20–165), *p<*0.001. Weight-for-age and height-for-age z-scores were stable over the period of the study. Basal hypertrigliceridemia and hiperchloresterolemia was present in 20 and 12%, respectively, and 8% (*p=*0.5) vs 25% (*p=*0.05) one year after starting the new regimen.

**Conclusions:**

Well tolerated and effective in our patients, DRV/r and RAL provide potentially good options for heavily pre-treated HIV-infected children. Regimens based on genotyping data were effective for children who had virological failure with multidrug-resistant HIV-1 infection. We observed a sustained antiviral response and improved immunologic indices in multidrug-resistant paediatric patients. Long-term follow-up is necessary to warrant the ARVT feasibility and sustainability.

## References

[1] Bandello K, Prieto L, Toledo M, Guillen S, Alvarez A, García Bermejo I (2012). Characteristics of human immunodeficiency virus-1 infected children receiving highly active antiretroviral therapy: a cross-sectional study. Ann Pediatr (Barc).

[2] Neely M, Kovacs A (2009). Managing treatment-experienced paediatric and adolescent HIV patients: role of darunavir. Ther Clin Risk Manag.

# Sub article: P029 Safety of tenofovir alafenamide in renal impairment

**DOI:** 10.7448/IAS.18.3.20160

**Published:** 2015-04-16

**Authors:** Jaime Andrade-Villanueva, Anton Pozniak, Jose Arribas, Samir Gupta, Frank Post, Anchalee Avihingsanon, Gordon Crofoot, Kenneth Lichtenstein, Moti Ramgopal, Ploenchan Chetchotisakd, Anita Silva, Marshall Fordyce

**Affiliations:** 1 HIV Unit, Hospital Civil de Guadalajara, Guadalajara, Mexico; 2 HIV Service, Chelsea and Westminster Hospital NHS Foundation Trust, London, UK; 3 Servicio de Medicina Interna, Hospital Universitario La Paz, Madrid, Spain; 4 Division of Infectious Diseases, Indiana University School of Medicine, Indianapolis, IN, USA; 5 Department of Renal Sciences, King's College Hospital NHS Foundation Trust, London, UK; 6 Faculty of Medicine, Chulalongkorn University, Bangkok, Thailand; 7 Thai Red Cross AIDS Research Center, Bangkok, Thailand; 8 Research Department, Crofoot Research, Houston, TX, USA; 9 Department of Medicine, National Jewish Health, Denver, CO, USA; 10 Midway Research Center, Fort Pierce, FL, USA; 11 Department of Medicine, Khon Kaen University, Khon Kaen, Thailand; 12 Public Health & Medical Affairs, Gilead Sciences Farmaceutica do Brasil, Sao Paulo, Brazil; 13 Clinical Research, Gilead Sciences, Foster City, CA, USA

## Abstract

**Introduction:**

Tenofovir (TFV) is renally eliminated, and the prodrug, tenofovir disoproxil fumarate (TDF), has been associated with renal toxicity and reduced bone mineral density (BMD) and must be dose adjusted in patients with estimated glomerular filtration rate (eGFR) <50 ml/min. Tenofovir alafenamide (TAF) is a novel prodrug of tenofovir (TFV), that is not renally eliminated and at clinical doses results in 90% lower plasma TFV levels as compared to TDF. The safety and efficacy of a once-daily single-tablet regimen of elvitegravir, cobicistat, emtricitabine and TAF (E/C/F/TAF) was assessed in HIV-1-positive patients with mild to moderate renal impairment.

**Materials and methods:**

Virologically suppressed adults with stable eGFR_C-G_ (Cockroft Gault) of 30 to 69 ml/min had their treatment switched from both TDF- and non-TDF-containing regimens to open-label E/C/F/TAF. Efficacy and safety data of Week 24 are described, including tests of renal function and BMD. Actual GFR (aGFR) was assessed with iohexol clearance in a subset of subjects.

**Results:**

Of 242 subjects enrolled and dosed, mean age was 58 years (range: 24 to 82), 18% were Black, 39% had hypertension and 14% had diabetes. Sixty-five percent were taking TDF-containing regimens prior to switch. At baseline, median eGFR_C-G_ was 55.6 ml/min (33% eGFR_C-G_ 30 to 49 ml/min). Ninety-five percent of subjects maintained HIV-1 VL <50 c/ml at Week 24 (FDA Snapshot). At Week 24, the median (Q1, Q3) change from baseline eGFR_C-G_ was 0.4 (−4.7, 4.5) ml/min, eGFR-cystatin C 3.8 (−4.8, 11.2) ml/min/1.73 m^2^, and aGFR (*n*=32, 68.8% TDF at baseline) was 0.1 (−4.3, 4.4) ml/min, indicating that GFR was not affected by E/C/F/TAF. Two subjects (0.8%) discontinued study drug for decreased GFR by eGFR_C-G_ and eGFR-cystatin C, neither with evidence of renal tubulopathy. The prevalence of clinically significant proteinuria (UPCR>200 mg/g) and albuminuria (UACR≥30 mg/g) decreased from 42 to 21% and 49 to 27%, respectively. Significant decreases in urine retinol binding protein to creatinine ratio, beta 2 microglobulin to creatinine ratio and fractional excretion of uric acid were observed (*p*<0.001 for all). Hip and spine BMD percentage change from baseline to Week 24 was 0.74% (−0.71, 2.03) and 1.27% (−0.44, 3.83) (median, IQR), respectively.

**Conclusions:**

These 24-week data support the virologic efficacy and renal and bone safety of once-daily single-tablet E/C/F/TAF for use in HIV+ patients with mild and moderate renal impairment (eGFR 30 to 69 ml/min). Switch to E/C/F/TAF was associated with no change in aGFR and with reductions in proteinuria.

# Sub article: P030 Renal and bone safety of tenofovir alafenamide versus tenofovir disoproxil fumarate

**DOI:** 10.7448/IAS.18.3.20161

**Published:** 2015-04-16

**Authors:** Edwin DeJesus, Paul Sax, Michael Yin, Frank Post, Shinichi Oka, Ellen Koenig, Benoit Trottier, Jaime Andrade-Villanueva, Huyen Cao, Julie Ryu, Marshall Fordyce

**Affiliations:** 1 Orlando Immunology Center, Orlando, FL, USA; 2 Division of Infectious Disease, Brigham and Women's Hospital, Boston, MA, USA; 3 Division of Infectious Diseases, Columbia University Medical Center, New York, NY, USA; 4 New York Presbyterian New York, NY, USA; 5 King's College Hospital, London, UK; 6 National Center for Global Health and Medicine Hospital, Tokyo, Japan; 7 Instituto Dominicano de Estudio Virologicos, Santo Domingo, Dominican Republic; 8 Clinique Medicale l'Actuel, Centre d'excellence VIH, Montreal, Canada; 9 HIV Unit, Hospital Civil de Guadalajara, Guadalajara, Mexico; 10 Clinical Research, Gilead Sciences, Foster City, CA, USA

## Abstract

**Introduction:**

Off-target renal and bone side effects may occur with tenofovir disoproxil fumarate (TDF) use. Compared with TDF, tenofovir alafenamide (TAF) results in significantly reduced plasma tenofovir (TFV) and may have less renal and bone toxicity.

**Materials and methods:**

Treatment-naïve HIV-1+ adults were randomized 1:1 to a single-tablet regimen of E/C/F/TAF or E/C/F/TDF once daily in two double-blind studies. Assessments for all subjects included measures of glomerular and proximal renal tubular function, and bone mineral density (BMD). Four pre-specified secondary safety endpoints were tested: serum creatinine, treatment-emergent proteinuria, spine and hip BMD; fasting lipid parameters were also collected. Week 48 off-target side effects data from both studies are described.

**Results:**

Combined, the two studies randomized and treated 1733 subjects. Plasma TFV was >90% lower (mean (% CV) AUCtau 297 (20) vs 3410 (25) ng*hr/ml) in the E/C/F/TAF arm, compared to the E/C/F/TDF arm. Serum creatinine (mean (SD) change: +0.08 (0.124) vs +0.11 (0.217) mg/dL, *p<*0.001), quantified proteinuria (UPCR, median (Q1, Q3) % change; −3 (−35, +43) vs +20 (−23, +76), *p<*0.001), albuminuria (UACR, median (Q1, Q3) % change; −5 (−33, +36) vs +7 (−27, +62), *p=*0.001), retinol binding protein (RBP:Cr, median (Q1, Q3) % change; +9 (−23, +49) vs +51 (+3, +133)), beta-2-microglobulin (β-2-Mg:Cr, median (Q1, Q3) % change; −32 (−57, +4) vs +24 (−34, +168)), and fractional excretion of phosphate (median (Q1, Q3) % change; +0.9 (−2.0, +4.5) vs +1.7 (−1.6, +5.3)), all favoured E/C/F/TAF. There were no cases of proximal tubulopathy in either arm. Mean (SD)% decrease in BMD was significantly less in the E/C/F/TAF arm for both lumbar spine (−1.30 (3.08) vs −2.86 (3.25), *p<*0.001) and total hip (−0.66 (3.26) vs −2.95 (3.41), *p<*0.001). Increases from baseline in bone turnover biomarkers (C-telopeptide and P1NP) and parathyroid hormone were significantly smaller in the E/C/F/TAF group compared with the E/C/F/TDF arm (*p<*0.001 for all). Increases in fasting lipid parameters (total cholesterol, HDL, direct LDL and triglycerides) were greater in the E/C/F/TAF arm (*p*<0.001 for all).

**Conclusions:**

Through 48 weeks, subjects receiving E/C/F/TAF had significantly better outcomes related to renal and bone health than those treated with E/C/F/TDF; lipid outcomes favoured E/C/F/TDF. Collectively these data demonstrate important safety benefits of TAF relative to TDF, especially given the ageing of the HIV population and the need for long-term treatment.

# Sub article: P031 Tenofovir alafenamide in a single-tablet regimen in initial HIV-1 therapy

**DOI:** 10.7448/IAS.18.3.20162

**Published:** 2015-04-16

**Authors:** Edwin DeJesus, David Wohl, Anton Pozniak, Melanie Thompson, Daniel Podzamczer, Jean-Michel Molina, Gordon Crofoot, Christian Callebaut, Hal Martin, Julie Ryu, Scott McCallister

**Affiliations:** 1 Orlando Immunology Center, Orlando, FL, USA; 2 AIDS Clinical Trials Unit, University of North Carolina, Chapel Hill, NC, USA; 3 Chelsea and Westminster NHS Trust, NHS Trust, London, UK; 4 AIDS Research Consortium of Atlanta, Atlanta, GA, USA; 5 Infectious Disease Service, Hospital Universitari de Bellvitge, Barcelona, Spain; 6 Department of Infectious Disease, Hôpital Saint Louis, Paris, France; 7 Crofoot Research, Medical Practice, Houston, TX, USA; 8 Clinical Research, Gilead Sciences, Foster City, CA, USA

## Abstract

**Introduction:**

Tenofovir alafenamide (TAF) is a novel tenofovir (TFV) prodrug that, when administered in the single-tablet regimen elvitegravir/cobicistat/emtricitabine/TAF (E/C/F/TAF), has >4-fold increase in intracellular TFV diphosphate and >90% lower plasma TFV levels compared to tenofovir disoproxil fumarate (TDF). Two phase 3 studies of identical design were conducted in distinct geographic areas comparing two single-tablet regimens, E/C/F/TAF and E/C/F/TDF, in treatment-naïve HIV-1+ adults.

**Materials and methods:**

Patients were randomized 1:1 to receive a single-tablet regimen of E/C/F/TAF or E/C/F/TDF once daily in two phase 3 double-blind studies. Primary end point was Week 48 virologic response by FDA Snapshot algorithm in a pre-specified analysis of the combined studies.

**Results:**

A total of 1733 subjects were randomized and treated: 15% women, 43% non-White, 23% viral load ≥100,000 copies/ml. Median baseline characteristics were: age 34 years, VL 4.58 log_10_ c/ml, and CD4 count 427 cells/µl. The primary objective was met, as E/C/F/TAF was non-inferior to E/C/F/TDF with 92 and 90%, respectively, having HIV RNA <50 copies/ml at Week 48 (difference +2%, 95% CI −0.7% to +4.7%, *p=*0.13). The rates of virologic success between E/C/F/TAF and E/C/F/TDF were similar across subgroups according to age, sex, race, baseline HIV-1 RNA level, baseline CD4 cell count, region (US vs ex-US) and study drug adherence. Mean change in CD4 count at Week 48 was 230 cells/µl in the E/C/F/TAF arm vs 211 cells/µl for E/C/F/TDF (*p=*0.02). Virologic failure with resistance occurred in 0.8% in the E/C/F/TAF arm and 0.6% on E/C/F/TDF. Treatment-related serious adverse events (AEs) were rare: E/C/F/TAF 0.3% (*n=*3), E/C/F/TDF 0.2% (*n=*2). There were no reports of proximal renal tubulopathy (including Fanconi syndrome) in either arm. No single AE led to discontinuation of more than one subject on E/C/F/TAF. Grade 2, 3 or 4 AEs occurring in ≥2% were: diarrhoea (3.3% vs 2.5%), nausea (2.2% vs 2.0%), headache (2.9% vs 2.1%), and URI (3.6% vs 3.1%) in the E/C/F/TAF and E/C/F/TDF arms, respectively.

**Conclusions:**

Through 48 weeks of treatment, high virologic response rates were seen in patients receiving E/C/F/TAF or E/C/F/TDF, and similar responses were seen across subgroups evaluated. Drug resistance was <1%. Both regimens were well tolerated, and no unique AEs associated with TAF occurred. These data support the use of E/C/F/TAF, the first TAF-based single-tablet regimen, as a potential new regimen for initial treatment of patients with HIV-1 infection.

# Sub article: P032 Dolutegravir versus raltegravir in ARV-experienced INI-naïve HIV+ adults: 48-week subgroup analysis of Latin American subjects in the SAILING study

**DOI:** 10.7448/IAS.18.3.20163

**Published:** 2015-04-16

**Authors:** Pedro Cahn, Jaime Andrade-Villanueva, Carlos Beltran, Tamara Newman, David Dorey, Dannae Brown, Isidoro Prudente, Sandy Griffith, Michael Aboud, Carlos Fernando de Oliveira

**Affiliations:** 1 Fundacion Huesped, Buenos Aires, Argentina; 2 CUCS, Hospital Civil de Guadalajara, Universidad de Guadalajara, Guadalajara, Mexico; 3 Hospital Barros Luco Trudeau, Santiago, Chile; 4 Instituto de Infectologia Emilio Ribas, Sao Paulo, Brazil; 5 GlaxoSmithKline, Mississauga, Canada; 6 ViiV Healthcare, Melbourne, Australia; 7 ViiV Healthcare, Santiago, Chile; 8 GlaxoSmithKline, Research Triangle Park, NC, USA; 9 ViiV Healthcare, London, UK; 10 PPD, Pharmacovigilance, Morrisville, NC, USA

## Abstract

**Introduction:**

The SAILING study compared the efficacy and tolerability of a dolutegravir (DTG) versus a raltegravir (RAL) containing regimen in antiretroviral-experienced, integrase-inhibitor-naïve adults with HIV-1. This subgroup analysis explores key efficacy and safety data for subjects enrolled in SAILING from Latin American (LA) countries.

**Methods:**

SAILING was a 48-week, Phase III, randomized, double-blind, active controlled, multinational, non-inferiority study, which compared DTG 50 mg once daily with RAL 400 mg twice daily, both in combination with investigator-selected background therapy (no more than two agents) in subjects with at least two class resistance. The primary endpoint was the proportion of subjects with HIV-1 RNA <50 copies/ml at Week 48 (FDA snapshot, modified intent-to-treat exposed population) [1]. This post hoc analysis includes subjects enrolled at centres from Argentina, Brazil, Chile and Mexico.

**Results:**

LA subjects accounted for 238/715 (33%) of the study population. Baseline characteristics and virologic response rates are shown in the table (Table 1). For the overall study, superiority of DTG compared to RAL was demonstrated at Week 48. For the LA sub group analysis, a higher proportion of subjects who received DTG compared to RAL achieved HIV-1 RNA <50 copies/ml in both the snapshot and treatment-related discontinuation equals failure analyses. There were fewer virologic non-responders in the DTG group (DTG, 20%; RAL 25%), and fewer subjects with protocol-defined virologic failure (DTG: *n=*8, 7%, RAL: *n=*17, 15%). There were few safety events leading to discontinuation from LA countries (DTG: 1 subject (<1%); RAL: 4 (3%)). The most common drug-related adverse events were diarrhoea (7% vs 5%) and nausea (2% vs 6%) for DTG vs RAL subjects, respectively.

**Conclusions:**

Consistent with the overall study results, DTG 50 mg once daily was effective and well tolerated in the subgroup of subjects enrolled in the SAILING study from Latin America. Low rates of virologic failure and discontinuations due to adverse events were observed in the DTG arm. DTG represents an important new therapeutic option for patients across Latin America and globally.

**Abstract P032–Table 1:** Summary of efficacy outcomes for overall and LA population at Week 48

Overall SAILING population [1]	DTG 50 mg once daily, *N=*354	RAL 400 mg BID, *N=*361
Number of responders at Week 48 (HIV-1 RNA<50 c/ml), *n/N* (%)	251/354 (71)	230/361 (64)
Adjusted difference in proportion (95% CI) (DTG-RAL)	7.4 (0.7, 14.2)	
Cochran-mantel-haenszel *P*-value	0.030	
**Latin America subgroup**	**DTG 50 mg once daily *N=*123**	**RAL 400 mg BID *N=*115**
Baseline HIV-1 RNA log_10_ c/ml, median (range)	4.17 (1.59, 6.16)	4.22 (1.59, 6.54)
Baseline CDC class C category, *n* (%)	70 (57)	51 (44)
Number of responders at Week 48 (HIV-1 RNA<50 c/ml), *n/N* (%)	91/123 (74)	78/115 (68)
Adjusted difference in proportion (95% CI) (DTG-RAL)	6.3 (−5.0, 17.7)	
Proportion (%) of subjects without treatment-related failure (TRDF), Kaplan-Meier estimate (95% CI)^a^	92.4 (86.0, 96.0)	81.9 (73.4, 87.9)
Difference in proportion (95% CI) (DTG-RAL)	10.5 (1.9, 19.1)	

a Treatment-related discontinuation equals failure (TRDF): protocol-defined virologic failure or withdrawal due to drug-related adverse event, safety stopping criteria; HIV-1 RNA greater than 50 c/ml not regarded as failure unless criteria for protocol-defined virologic failure were met.

## References

[1] Cahn P, Pozniak AL, Mingrone H, Shuldyakov A, Brites C, Andrade-Villanueva JF (2013). Dolutegravir versus raltegravir in antiretroviral-experienced integrase-inhibitor-naïve adults with HIV: week 48 results from the randomised, double-blind, non-inferiority SAILING study. Lancet.

# Sub article: P033 Ageing in Latin America; results of a large collaborative study group

**DOI:** 10.7448/IAS.18.3.20175

**Published:** 2015-04-16

**Authors:** Carlos Beltran, Omar Sued, Ana Celi, Paola Castillo, Monica Thormann, Olga Lopez, Carolina Chahin, Carlos Gallo, Carlos Perez, Alejandro Afani

**Affiliations:** 1 Department of Infectious Diseases, Latin American Workshop Study Group, Santiago, Chile; 2 Department of Infectious Diseases, Hospital Juan A. Fernandez, Buenos Aires, Argentina; 3 Department of Infectious Diseases, Hospital de Especialidades Fuerzas Armadas, Quito, Ecuador; 4 Department of Infectious Diseases, Hospital Teodoro Maldonado, Guayaquil, Ecuador; 5 Department of Infectious Diseases, Hospital Salvador B Gautier, Santo Domingo, Dominican Republic; 6 Department of Infectious Diseases, Hospital Ernesto Torres Galdames, Iquique, Chile; 7 Department of Infectious Diseases, Hospital Herna Henriquez Aravena, Temuco, Chile; 8 Department of Infectious Diseases, Hospital Juan Noe, Arica, Chile; 9 Department of Infectious Diseases, Hospital Clínico Pontificia Universidad Católica, Santiago, Chile; 10 Department of Immunology, Hospital Clínico Universidad de Chile, Santiago, Chile

## Abstract

**Introduction:**

By 2015, up to 50% of patients linked to care may be over 50 years old in some countries. Limited research has explored this issue in Latin American countries. The Latin American Study Group is a collaborative workshop of 19 HIV care centres from six countries including data of 30,524 HIV-positive patients. We compared age and gender distribution in HIV-positive population in Latin American countries and the impact of HIV acquisition at older ages.

**Materials and methods:**

A cross-sectional study was done between September 2014 and January 2015 in Peru, Argentina, Chile, Colombia, Ecuador and Dominican Republic. Data from 24,384 out of 30,524 patients with at least one visit in the last 12 months are presented. Age and gender distribution in newly diagnosed patients with HIV infection was also collected. Descriptive and analytical statistics were used to compare differences in age and gender distribution among centres in patients in active care and in those recently diagnosed.

**Results:**

Of 24,384 patients, 5662 (23.2%) were women; 60.3% of active patients are 30 to 49 years old, 17.4% are younger than 30 and only 22.3% are older than 50 years (1.4% older than 70) without gender differences in age distribution. Nevertheless, patients older than 50 years ranged from 16.3% in Ecuador to 33.7% in Dominican Republic (*p<*0.001). Among 2960 newly diagnosed patients, only 15.3% are older than 50 years old ranging from 14.6% in Chile to 28.9% in Dominican Republic (*p<*0.001).

**Conclusions:**

To the best of our knowledge, this is the largest report on HIV and ageing in Latin America. HIV-positive patients in active control are younger than those reported in Europe and USA. Less than 25% of patients linked to care and only one out of six newly diagnosed patients are older than 50 years old without differences in ageing by gender but with regional differences that warrant further research.

# Sub article: P034 Darunavir-containing deep salvage regimens in routine clinical practice: a stewardship strategy

**DOI:** 10.7448/IAS.18.3.20176

**Published:** 2015-04-16

**Authors:** Juan Calva, Carolina Lara-Sánchez, Pedro Aguilar-Salinas, Mauricio Ostrosky-Frid

**Affiliations:** Department of Infectious Diseases, Instituto Nacional de Ciencias Médicas y Nutrición “Salvador Zubirán”, Mexico City, Mexico

## Abstract

**Introduction:**

This study aims to assess the virologic and immunologic effects of a ritonavir-boosted darunavir (DRV/r)-containing salvage regimens recommended to physicians by an antiretroviral (ARV) therapy peer-advisory committee, in patients with extensive ARV treatment experience and multiple treatment failures.

**Materials and methods:**

Nationwide, HIV-clinic-based cohort study was conducted in Mexico. Eligible patients were HIV-positive adults who had experienced the virologic failure of at least two prior ARV regimens and had detectable viremia while currently being treated; their physicians had received a therapeutic advice regarding the DRV/r-containing salvage regimen, by a panel of experts (intervention). Median (Md) follow-up time was 47 months (interquartile range (IQR)=38 to 57 months) with periodical plasma HIV-RNA level (pVL) and blood CD4+ T-cell count (CD4+) measurements. Outcomes were cumulative incidence (Kaplan-Meier survival analysis) and risk factors (Cox proportional hazards regression modelling) of loss of virologic response (LVR) (pVL of less than 200 copies per ml, followed by levels above this threshold) and change of CD4+.

**Results:**

A total of 380 patients were followed up. Md ARV therapy exposure=12 years; Md prior regimens=4; Md major protease inhibitor-resistance-associated mutations (mPI-RAM)=3; Md DRV-RAM=1. The probabilities of LVR were 4.5%, 5.6%, 6.7%, 8% and 11.7% at the 12-, 24-, 36-, 48- and 60-month follow-up assessments, respectively. Of the 346 patients who achieved virologic response (VR), Md increase in CD4+ was 206 cell/ml (IQR=79 to 341 cells/ml); *p<*0.001. Patients aged <40 years were more likely to lose VR (hazard ratio=2.6; 95% CI: 1.3 to 5.6; *p=*0.01). Nadir of CD4+, number of mPI- and of DRV-RAM and genotypic sensitivity score (GSS) (<3 vs ≥3) of the regimen were not associated with failure.

**Conclusions:**

Our intervention aimed at avoiding functional monotherapy with DRV/r in deep salvage therapy led to a high rate of long-lasting VR and immune reconstitution in heavily ARV-experienced patients. LVR is associated with young age (as a possible surrogate of lack of adherence) and is independent of nadir of CD4+, basal mPI- and DRV-RAM. It seems that, under optimal patient's compliance, a DRV/r-containing regimen with at least 2.5 fully active drugs is highly effective in routine clinical practice.

# Sub article: AUTHOR INDEX

**Published:** 2015-04-16

## A

**Table:** 

Aboud, M	P032
Abreu, M	P020
Afani, A	P033
Aguilar-Salinas, P	P034
Allavena, C	P017
Alonso González, M	O321*
Álvarez, J	P014
Andrade-Villanueva, J	P009*, P018, P029*, P030, P032
Andre-Garnier, E	P017
Arribas, J	P029
Avihingsanon, A	P029
Avila, I	P023
Avila-Rios, S	O331*

## B

**Table:** 

Batra, J	P026*
Bautista-Arredondo, S	KL12
Belaunzarán-Zamudio, P	O112, O123, O332*, O333
Beltran, C	P032, P033*
Bennett, S	P026
Bernaud, C	P017*
Bertozzi, S	KL12*
Beyrer, C	O111*
Bhatti, L	P011
Brainard, D	O322
Brown, D	P032
Brugger, K	P024
Bugarin, G	O113*

## C

**Table:** 

Cahn, P	P016, P032*
Callebaut, C	P031
Calva, J	P034*
Campo, R	O222*
Cao, H	P030
Carmona, JA	P015
Caro-Vega, Y	O123, O333*
Carriquiry, G	O123
Casillas-Rodríguez, J	O112, O332, P004
Cassetti, I	O113
Castillo, P	P033
Castillo-Mancilla, J	O214*
Castro, J	P008
Celi, A	P033
César, C	O333
Chahin, C	P033
Chanqueo, L	P024*
Cheng, A	P026
Chetchotisakd, P	P029
Conde-Gonzalez, CJ	P005
Cortés, C	O123, O333
Crabtree Ramírez, B	O123*, O333
Crofoot, G	P029, P031
Cruz, J	O112, P007, P021, P022
Cruz Palacios, C	P010

## D

**Table:** 

de Jesus Herrera-Castillo, C	P027*
de Jesus Cabrera López, T	P010*
de Oliveira, CF	P032
del Rio, C	KL32*
DeJesus, E	P030*, P031*
Díaz, S	O332, P010
Dieterich, D	O322
Dorey, D	P032
Duen̈as Dominguez, RA	P012*

## E

**Table:** 

Eron, J	P011
Escobar, D	P015

## F

**Table:** 

Ferreyra, D	P007, P021, P022
Fink, V	O123
Flores-Ruiz, E	P028
Foradori, I	P001
Fordyce, M	P029, P030
Frola, C	P016

## G

**Table:** 

Gaggar, A	O322
Galarraga, O	P005
Gallo, C	P033
García-Morales, C	O331
Gaur, A	P026
Godinez Hernández, SI	P014*
Gomez, N	P002, P003
Gonçalves, C	P020*
Gonzalez, A	P005
Gonzalez, A	P015
Gonzalez, JC	P015*
González-Hernández, L	P009, P018*
González-Rodríguez, A	O112, O332, P010
Gras Allain, N	P004, P005*
Greatorex, J	P024
Griffith, S	P032
Grinsztejn, B	O123, O333
Guelfand, L	P016
Gulick, R	KL31*
Gupta, S	P029

## H

**Table:** 

Hall, N	P017
Hernandez, AM	P006
Hernandez, G	P022
Hidalgo, J	P013
Hirata, H	P007*, P021
Hu, Y	P011
Huerta-García, G	P028

## I

**Table:** 

Illescas, R	P013
Ivalo, S	P001

## J

**Table:** 

Jayathilake, K	O123
Jimenez Guevara, EF	P014
Jovelin, T	P017

## K

**Table:** 

Kizito, H	P026
Koenig, E	P002, P030
Kuritzkes, D	O211*

## L

**Table:** 

Langarica Naves, E	P010
Lara-Sánchez, C	P034
Lawson, E	P026
Lederman, M	KL11*
León Guerrero, EM	P014
Lemos, JA	P008
Lichtenstein, K	P029
Lima-Verde, R	P008
Lira, R	P027
Llamas-Covarrubias, M	P009, P018
Lockhart, P	P002, P003
Lopez, O	P033
Lopez Segura, M	P006*
Lorenzana, I	O331
Losso, M	P001
Luz, P	O333

## M

**Table:** 

Macías-González, I	O332
Madruga, JV	O113
Magis Rodríguez, C	P019
Malanca, A	P001
Malpartida, O	P013*
Manzanero, M	O331
Maquera, J	P013
Martin, H	P024
Martin, H	P031
Martínez-Ayala, P	P009
Martínez-Olmedo, J	P018
Martinez-Tristani, M	O113
Massetto, B	O322
Mata-Marín, JA	P028
Mayer, K	P005
McCallister, S	P031
McGovern, B	P011
McGowan, C	O123, O333
McHutchison, J	O322
Mejía-Cordero, F	O333
Mejía-Villatoro, C	O331
Mendez, J	P020
Mercado-Nuñez, E	P018
Molina, J-M	O322, P031
Morrier, M	P017
Mosqueda, JL	P006, P014
Munoz, G	P023*

## N

**Table:** 

Naggie, S	O322
Newman, T	P032
Ni, L	O322

## O

**Table:** 

Oka, S	P030
Oliveira, E	P008
Oliveira-Filho, A	P008*
Operario, D	P005
Orkin, C	O322
Ostrosky-Frid, M	P034

## P

**Table:** 

Padgett, D	O123, O333
Palou, E	O331
Pape, JW	O212*, O333
Pascale, J	O331
Paulino, R	P002*, P003*
Peregrino-Bejarano, L	P028
Pereira, P	P008
Perez, C	P033
Perez, G	P013
Pérez, H	P016
Perre, P	P017
Person, A	O123
Pineau, S	P017
Pineau, S	P017
Pinto, L	P008
Podzamczer, D	P031
Ponce-Herrera, D	P009
Porras-Cortes, G	O331
Post, F	P029, P030
Pozniak, A	O311*, P029, P031
Prasitsuebsai, W	P026
Prudente, I	P032
Puello, A	P002, P003
Puoti, M	O322

## Q

**Table:** 

Quirk, E	P026

## R

**Table:** 

Raffi, F	P017
Rakhmanina, N	P026
Ramgopal, M	P029
Ramos-Alamillo, U	O332, P010, P012
Reyes-Terán, G	O331
Rivera, C	O113
Rocabert, C	P007, P021, P022
Rockstroh, J	O322
Rodallec, A	P017
Rodríguez, A	P027
Rodríguez, JC	P004
Rodríguez, L	P013
Rodriguez, R	P013
Rodriguez, V	P007, P021, P022*
Rodriguez-Torres, M	O322
Rojas-Montes, O	P027
Román, E	O112*
Ruane, P	P011
Ruiz, V	P012
Ryu, J	P030, P031

## S

**Table:** 

Saavedra, B	P005
Sadik, A	P024
Santana, J	O322*
Sarmento e Castro, R	P020
Sawada, L	P008
Sax, P	P030
Shao, Y	P026
Shepherd, B	O123, O333
Shulman, N	P011
Sierra-Madero, J	O112, O123, O213*, O333
Silva, A	P026, P029
Silva, AR	P020
Solorzano-Santos, F	P028
Soria-Rodríguez, R	P009, P018
Sosa-Rubi, S	KL12, P005
Soto-Ramírez, L	P023
Stankievich, E	P001*
Sterling, TR	O122*
Straminsky, F	P016
Subramanian, M	O322
Sued, O	O121*, P016*, P033
Sulkowski, M	O322, P011
Svarovskaia, E	O322

## T

**Table:** 

Tapia-Trejo, D	O331
Thompson, M	P031
Thormann, M	P033
Trinh, R	P011*
Trottier, B	P030

## V

**Table:** 

Valenzuela Lara, M	P019*
Vallinoto, A	P008
van Wyk, J	O113
Vargas González, H	P004*
Vasquez, P	O113
Vazquez-Rosales, JG	P027, P028*
Vega-Ramirez, H	O112, P007, P021*, P022
Volkow, P	O221*

## W

**Table:** 

Wang, C	P011
Wohl, D	P031
Wolff, M	O123
Wyles, D	P011

## Y

**Table:** 

Yin, M	P030

## Z

**Table:** 

Zhang, H	P024
Zuñiga-Quiñonez, S	P009
